# Lhx1/5 control dendritogenesis and spine morphogenesis of Purkinje cells via regulation of Espin

**DOI:** 10.1038/ncomms15079

**Published:** 2017-05-18

**Authors:** Nga Chu Lui, Wing Yip Tam, Caiji Gao, Jian-Dong Huang, Chi Chiu Wang, Liwen Jiang, Wing Ho Yung, Kin Ming Kwan

**Affiliations:** 1 School of Life Sciences, The Chinese University of Hong Kong, Shatin, Hong Kong, China; 2 School of Biomedical Sciences, University of Hong Kong, Pokfulam, Hong Kong, China; 3 Department of Obstetrics and Gynaecology, The Chinese University of Hong Kong, Shatin, Hong Kong, China; 4 School of Biomedical Sciences, The Chinese University of Hong Kong, Shatin, Hong Kong, China; 5 Li Ka Shing Institute of Health Sciences, The Chinese University of Hong Kong, Shatin, Hong Kong, China; 6 Center for Cell & Developmental Biology, The Chinese University of Hong Kong, Shatin, Hong Kong, China; 7 Partner State Key Laboratory of Agrobiotechnology (CUHK), The Chinese University of Hong Kong, Shatin, Hong Kong, China; 8 Gerald Choa Neuroscience Centre, The Chinese University of Hong Kong, Shatin, Hong Kong, China

## Abstract

In the cerebellar cortex, Purkinje cells (PCs) receive signals from different inputs through their extensively branched dendrites and serve as an integration centre. Defects in the dendritic development of PCs thus disrupt cerebellar circuitry and cause ataxia. Here we report that specific inactivation of both *Lhx1* and *Lhx5* in postnatal PCs results in ataxic mutant mice with abnormal dendritic development. The PCs in the mutants have reduced expression of Espin, an F-actin cytoskeleton regulator. We show that Espin expression is transcriptionally activated by Lhx1/5. Downregulation of Espin leads to F-actin mislocalization, thereby impairing dendritogenesis and dendritic spine maturation in the PCs. The mutant PCs therefore fail to form proper synapses and show aberrant electrophysiological properties. By overexpressing Espin, we can successfully rescue the defects in the mutant PCs. Our findings suggest that Lhx1/5, through regulating Espin expression, control dendritogenesis and spine morphogenesis in postnatal PCs.

## Abstract (web summary)

Purkinje cells (PCs) receive signals from different inputs through their extensively branched dendrites and dysregulation of this process leads to ataxia and other diseases. Here the authors show that the LIM-homeodomain transcription factors Lhx1 and Lhx5 govern dendritogenesis and dendritic spine morphogenesis in postnatal PCs through regulating Espin expression.

The cerebellum is the motor coordination centre that helps control muscle movement and maintains body balance. The Purkinje cell (PC) is a key component in cerebellar neuronal circuitry as it innervates with all cerebellar neurons either directly or indirectly^1^,^2^. After processing different inputs, the PC, the sole output neuron in the cerebellar cortex, sends signals to other parts of the brain via deep cerebellar nuclei. Therefore, the final cerebellum outputs and thus the execution of motor activities are primarily instructed by the firing patterns of PCs^3^,^4^,^5^. Due to its pivotal roles, abnormal development of PCs causes ataxia, a neurological disease characterized by tremor, loss of body balance and coordination^6^,^7^,^8^.

Previously, we identified two Lin11-Isl1-Mec3(LIM)-homeodomain genes, *Lhx1* and *Lhx5*, that are required for the early differentiation of PCs in embryonic mouse cerebellum^9^. These two genes share high sequence similarities at both messenger RNA (mRNA) and protein levels and are functionally redundant in controlling the early differentiation of PCs. Genes controlling embryonic development may not play a role in later postnatal development and their expression diminishes after birth. However, *Lhx1*/*5* is persistently expressed throughout postnatal and adult stages^10^ in differentiated PCs, implying that they may have other functional roles in PC postnatal development.

The most important process in the postnatal PCs is dendritic development^11^,^12^. To receive numerous presynaptic signals, the PC develops an extensively branched dendritic tree that is crucial for PC function and thus its malformation causes ataxia^13^,^14^. Different extrinsic factors have been shown to contribute in the dendritic development of PCs^15^. However, these dendrites can still develop in the absence of extrinsic factors, suggesting that intrinsic factors also play an important role^16^. Therefore, we hypothesize that *Lhx1/5* may be the intrinsic factors required for the dendritic development of PCs.

To specifically address the role of *Lhx1/5* in postnatal PCs, we generate *Lhx1/5* double conditional knockout (DKO) mice, in which *Lhx1/5* are specifically inactivated in all postnatal PCs. The DKO mutants display severe but nonprogressive motor deficits. A novel actin regulatory protein, Espin, is found to be downregulated in the PCs of DKO mutants and its gene (*Espn*) is confirmed to be transcriptionally activated by Lhx1/5. Downregulation of Espin results in altered F-actin localization in the dendrites of PCs and also impaired dendritogenesis and spine morphogenesis. Consequently, PCs fail to properly innervate with their presynaptic inputs and exhibit abnormal electrophysiological properties. Our findings provide evidences for a novel pathway for the control of dendritic development in which Lhx1/5, through regulating Espin expression, control F-actin localization in the dendrites of PCs and govern dendritogenesis and spine morphogenesis of PCs in the postnatal cerebellum.

## Results

### *Lhx1/5* DKO mutants have reduced PC dendritic length

To circumvent early lethality caused by *Lhx1-* or *Lhx5-*null mutation^17^,^18^, we generated a conditional null (*fx*) allele of *Lhx5* (Supplementary Fig. 1a–c), while the generation of the *Lhx1 fx* allele has been previously described^19^. Mice homozygous for the *Lhx5 fx* allele were fertile and phenotypically normal. To determine if the *Lhx5 fx* allele could reproduce the *Lhx5-*null phenotype, we crossed *Lhx5*^*fx/fx*^ mice with *Zp3-Cre* mice^20^ to generate a germline deletion of *Lhx5* (that is, *Lhx5*^Δ/Δ^). As expected, dentate gyrus and Ammon's horn of the hippocampus were absent in *Lhx5*^Δ/Δ^ mice (Supplementary Fig. 1d), a phenocopy of the previously described *Lhx5*-null mutant^18^,^21^. This proves that the *Lhx5 fx* allele is functional in response to Cre recombination.

**Figure 1:**
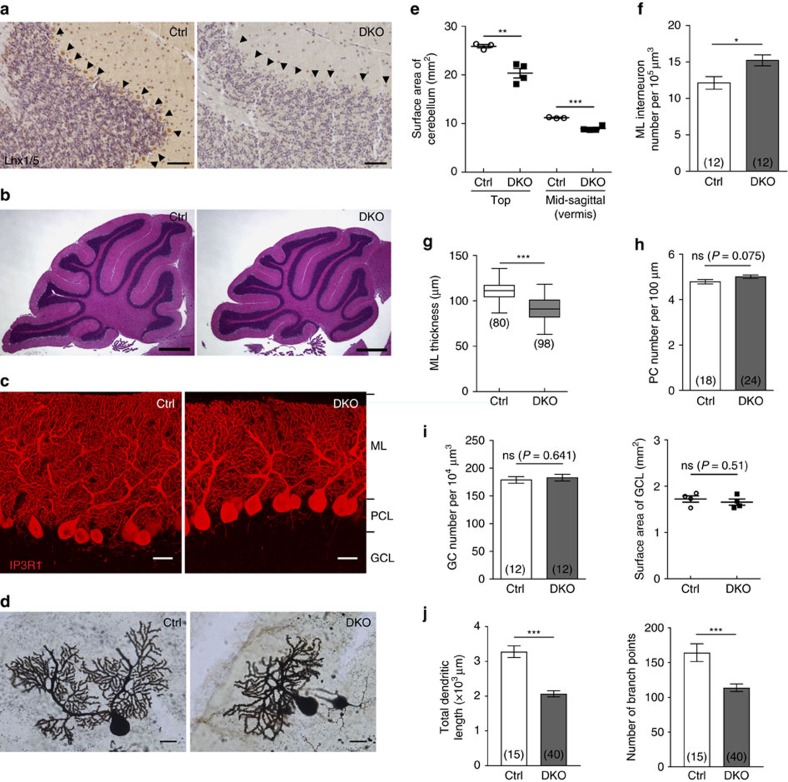
Normal cytoarchitecture of cerebellum but reduced length of PC dendrites in *Lhx1/5* DKO mutants. (**a**) Immunostaining of Lhx1/5 in the PCs (arrowheads) of the control (Ctrl) (left) and the DKO mutant (right). Scale bars, 50 μm. (**b**) Haematoxylin and eosin (H&E) staining showing the cytoarchitecture and foliation of the cerebellum of the control (left) and the DKO mutant (right). Scale bars, 500 μm. (**c**) Immunostaining of IP3R1 showing the PCs of the control (left) and the DKO mutant (right). ML, molecular layer; PCL, Purkinje cell layer; GCL, granule cell layer. Scale bars, 20 μm. All immunostaining or staining experiments were replicated at least three times with at least three mice per genotype. (**d**) Golgi-impregnated PCs of the control (Ctrl) (left) and the DKO mutant (right). Scale bars, 20 μm. (**e**) Scatter plot showing the size of cerebellum decreased in the DKO mutants (measured by the surface area of cerebellum at different views as shown in Supplementary Fig. 2b). (**f**) Bar graph showing that the density of interneurons in ML increased in the DKO mutants. (**g**) Box plot showing that the DKO mutant cerebella had reduced molecular layer thickness in lobule IV–V. Boxes indicate the mean (middle line) and 25 to 75% range, while whiskers indicate maximum and minimum values. (**h**) Bar graph showing the density of PCs was comparable between the mutants and the controls. (**i**) Bar graph (left) showing the density of GCs and scatter plot (right) showing the surface area of GCL (measured by the GCL area in H&E staining shown in (**b**)) was comparable between the mutants and the controls. (**j**) Bar graphs showing the total dendritic length (left) and the branching number (right) of the Golgi-impregnated PCs. Values in the brackets indicate the number of sections analysed. For all scatter plots, the bars indicate the mean values and each point represents a mouse cerebellum. For each measurement, at least three mice were analysed per group; *t*-test; ns, not significant; *P<0.05, **P<0.01 and ***P<0.001.

To study the functional roles of *Lhx1*/*5* in postnatal PCs, we generated *Lhx1/5* DKO mutants by crossing *Lhx1/5 fx* mice with *Pcp2*-*Cre* transgenic mice^22^, which specifically express Cre recombinase in PCs starting from postnatal day (P) 2 (Supplementary Fig. 2a) and in all PCs by P21 (ref. 22). To confirm the effectivity of the knockout of *Lhx1/5* in the PCs of the DKO mutants (*Pcp2-Cre; Lhx1*^*fx/fx*^*; Lhx5*^*fx/fx*^), we performed immunostaining using a LIM1 (Lhx1) antibody that also crossreacted with Lhx5 due to high protein sequence similarities. A positive signal was only detected in the PCs of the controls (*Pcp2-Cre; Lhx1*^*fx/+*^*; Lhx5*^*fx/fx*^ or *Pcp2-Cre; Lhx1*^*fx/fx*^*; Lhx5*^*fx/+*^) but not in the mutants (Fig. 1a), suggesting that *Lhx1*/*5* were specifically inactivated in the mutant PCs.

The cerebellum of the DKO mutants had normal foliation and cytoarchitecture when compared with the controls (Fig. 1b). However, the size of the mutant cerebellum decreased by 19.9–21.3% (Fig. 1e) because the molecular layer (ML) thickness decreased by 17.3% (Fig. 1c,g) due to the reduced dendritic length and branching of the mutant PCs (Fig. 1d,j). Since the ML thickness decreased, interneurons located in the ML (marked by parvalbumin, Supplementary Fig. 2d) of the DKO mutant were more tightly packed, resulting in an increase in the ML interneuron density (Fig. 1f). On the other hand, the morphology of GABAergeric interneurons (labelled by Pax2) and Bergmann glial fibres (labelled by glial fibrillary acidic protein) in the DKO mutants were comparable with the controls (Supplementary Fig. 2c). No significant differences were observed in the number of granule cells (marked by Pax6, Supplementary Fig. 2d, and Fig. 1i). Unexpectedly, comparable PC density was observed in the controls and in the DKO mutants (Fig. 1h). This implies that Lhx1/5 are required for the dendritic development of PCs, but not for the maintenance of postnatal PCs.

### *Lhx1/5* DKO mutants display motor deficits

**Figure 2:**
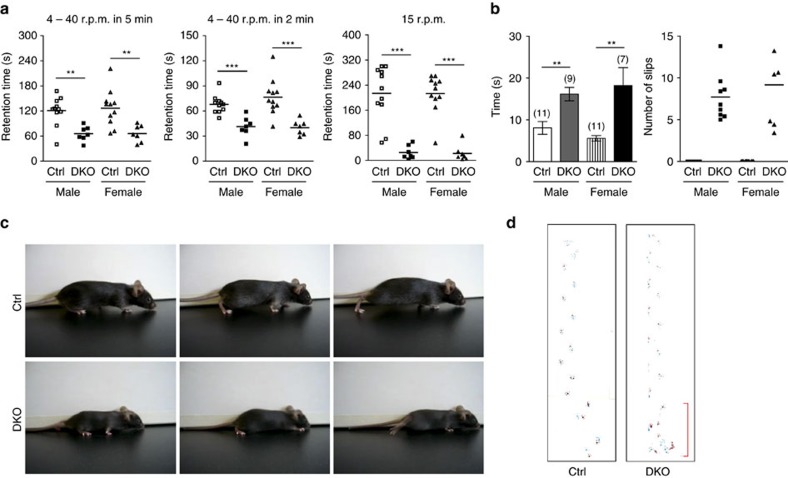
*Lhx1/5* DKO mutants display motor deficits. (**a**) Scatter plots showing differences in retention time before falling from an accelerating rotarod from 4 to 40 r.p.m. in 5 min (left) or 2 min (middle) and stationary rotarod at 15 r.p.m. (right) between the controls (*n*=22 mice) and the DKO mutants (*n*=14 mice). Bars in the scatter plots represent the mean values. (**b**) Graphs showing differences in time to complete running on an elevated balance beam (left) and number of missteps (right) between the controls and the DKO mutants. Brackets show the number of the control and the DKO mutant mice tested; *t*-test; **P<0.01 and ***P<0.001. (**c**) Representative photos showing the walking behaviour of a control (upper panels) and a DKO mutant (lower panels). At least three mice per group were tested for their walking behaviour. (**d**) Example of paw prints acquired during footprint analysis. The DKO mutant footprint showed some tottering steps (red bracket) during initiation of locomotion. In all, 21 control mice and 17 mutant mice were analysed.

Inactivation of *Lhx1/5* in postnatal PCs is not life threatening and all DKO mutants survive to adulthood. However, the mutants displayed noticeable ataxic locomotion beginning at ∼3 weeks after birth. Body tremor became prominent when the mutants were placed on a narrow ledge (Supplementary Movies 1 and 2). To quantitatively analyse their motor abilities, 2-month-old mice were subjected to rotarod and elevated balance beam tests^23^. The DKO mutants performed significantly poorer than the controls on both accelerating and stationary rods (Fig. 2a). Furthermore, the mutants took a significantly longer time to finish the elevated beam with more missteps (Fig. 2b). In gait analysis, the DKO mutants crawled with a lowered pelvis with their abdomen touching the ground and their hindlimbs pointed away from the body during locomotion (duck feet) (Fig. 2c and Supplementary Movie 3 and 4). In addition, their first few steps were tottering, which was also detected in their footprint patterns (Fig. 2d). However, apart from these tottering steps, there were no significant differences in stride length and forelimb–hindlimb overlapping between the mutants and the controls (Supplementary Fig. 3a). All of these abnormal gaits were not observed in the controls with a functional allele of either *Lhx1* or *Lhx5*, indicating that Lhx1 and Lhx5 are functionally redundant in postnatal PCs. Comparable motor performances between male and female DKO mutants suggests that the defects are not gender dependent. We also compared the rotarod performance between 6-month-old mice and 2-month-old mutants and their motor performances did not deteriorate progressively (Supplementary Fig. 3b–d). Together, the results suggest that inactivation of *Lhx1*/*5* in postnatal PCs leads to nonprogressive ataxia and motor deficits.

### Lhx1/5 transcriptionally regulate Espin expression

**Figure 3:**
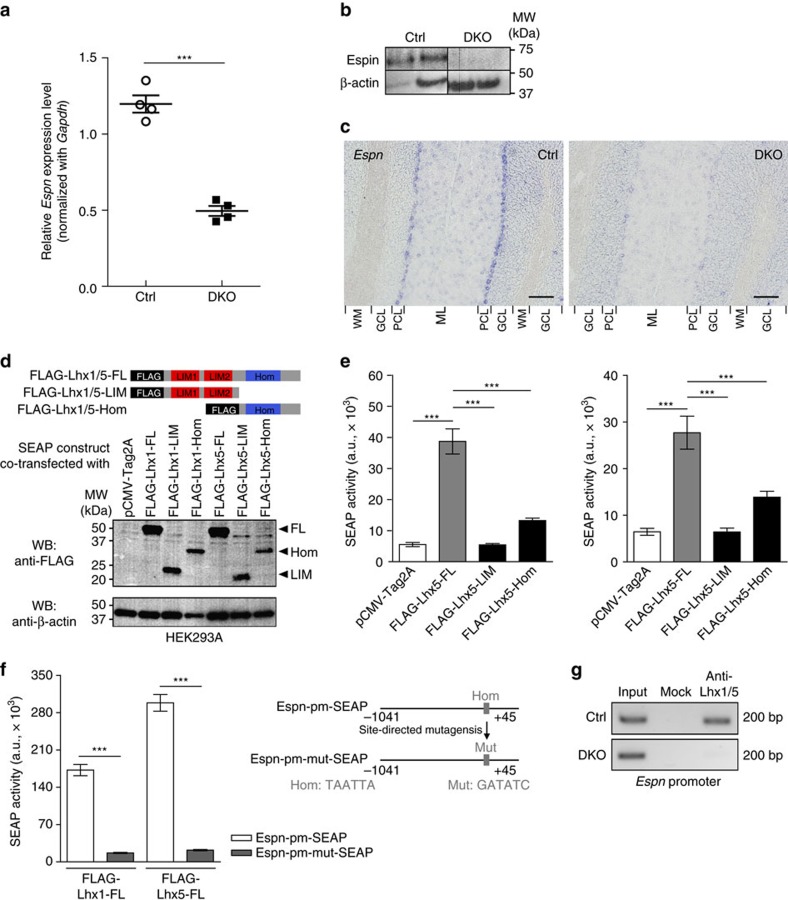
Lhx1/5 transcriptionally regulate *Espn*. (**a**) Real-time quantitative PCR showing the relative *Espn* expression level in the adult DKO mutant cerebellum when compared with the control cerebellum; *n*=4 mice per group; *t-*test; ***P<0.001. (**b**) Western blots showing the cerebella of the DKO mutants had lower Espin level than the controls. The experiment was repeated twice, seven mice for each genotype. (**c**) *In situ* hybridization for *Espn* transcripts showing the PCs of the DKO mutants (right) had a lower *Espn* expression than the controls (left). The experiment was repeated three times with three mice per genotype. ML, molecular layer; PCL, Purkinje cell layer; GCL, granule cell layer; WM, white matter. Scale bars, 100 μm. (**d**) Schematic illustrations showing different forms of FLAG-tagged Lhx1/5 proteins. Western blots showing the coexpression of Lhx1/5 proteins with SEAP. FL, full-length; LIM, LIM domains; Hom, homeodomain. (**e**) Bar graphs showing the differences in SEAP activity under the control of the *Espn* promoter (Espn-pm-SEAP) when different forms of Lhx1 (left) and Lhx5 (right) proteins were coexpressed. Analysis of variance (ANOVA); ***P<0.001. (**f**) Schematic illustrations showing the SEAP plasmid containing an *Espn* promoter with mutation (Mut) in the binding site of Lhx1/5 homeodomain (Hom). Bar graphs reveal the differences in the SEAP activity with and without the mutation in the *Espn* promoter. For all SEAP assays, at least three independent replications were performed and *n*=9 for each group of experiments; *t*-test; ***P<0.001. (**g**) Chromatin immunoprecipitation (ChIP)-PCR showing the *in vivo* binding of Lhx1/5 on *Espn* promoter in the controls (top) but not in the DKO mutants (bottom); *n*=2 mouse cerebella pooled for each group. The ChIP experiment was repeated three times, each with two different mouse cerebella per group.

To dissect the molecular mechanisms leading to the defects, the gene expression profiles of the DKO mutants and the controls were compared by microarray analysis. With a setting of fivefold changes in expression and *P* value of <0.05 (*n*=3 per group, one-way analysis of variance), 78 genes passed the criterion, with 45 upregulated and 33 downregulated (Supplementary Tables 1 and 2). As previous studies suggest actin regulatory proteins play critical roles in dendritic development^24^,^25^,^26^,^27^, here we specifically investigated a gene, *Espn*, that encodes a novel actin regulatory protein called Espin. To validate the microarray results, we checked Espin expression in the DKO mutant cerebella by real-time quantitative PCR and western blot analysis and confirmed that Espin was significantly downregulated at both mRNA and protein levels (Fig. 3a,b). Since *Espn* was specifically expressed in PCs only^28^ in the mouse cerebellum, the downregulation of *Espn* in cerebellum should be only due to reduced *Espn* expression in PCs. Using *in situ* hybridization, we detected *Espn* mRNA in cerebellum sections and found that *Espn* expression was specifically reduced in the mutant PCs but remained normal in the control PCs (Fig. 3c).

Since Lhx1/5 are transcription factors^29^, it is likely that the transcription of *Espn* is directly under the control of Lhx1/5. To test whether Lhx1/5 acts on the *Espn* promoter, a secreted alkaline phosphatase (SEAP) assay was performed by co-transfection with a SEAP plasmid containing the *Espn* promoter (Espn-pm-SEAP) (Supplementary Fig. 4) and Lhx1/5 expression plasmids into HEK293A cells (Fig. 3d). SEAP would be expressed when the *Espn* promoter was activated. The results revealed that both Lhx1 and Lhx5 full-length (FL) protein could individually strongly induce SEAP expression, while truncated Lhx1/5 (LIM/Hom) proteins and the control (pCMV-Tag2A) could not (Fig. 3e). To check whether the *Espn* promoter was directly activated by Lhx1/5, we mutated the potential binding site of the Lhx1/5 homeodomain^30^ on the *Espn* promoter to generate a mutated construct of Espn-pm-SEAP (that is, Espn-pm-mut-SEAP) (Fig. 3f). With this mutation, Lhx1/5 could no longer trigger SEAP expression (Fig. 3f), implying that Lhx1/5 can bind directly to the *Espn* promoter for transcriptional activation.

To demonstrate binding of Lhx1/5 to *Espn* promoter *in vivo*, chromatin immunoprecipitation was performed with cerebellum chromatin extracts from the controls and the DKO mutants. The *Espn* promoter fragment was immunoprecipitated in the control chromatin reacted with anti-Lhx1/5, but not in the mutant chromatin (Fig. 3g). Binding between Lhx1/5 and the *Espn* promoter was lost in the mutant cerebellum due to knockout of *Lhx1/5* in PCs. This provides evidences that Lhx1/5 can directly regulate *Espn* expression in postnatal PCs *in vivo*.

### Knockdown of *Espn* disrupts PC dendritic development

**Figure 4:**
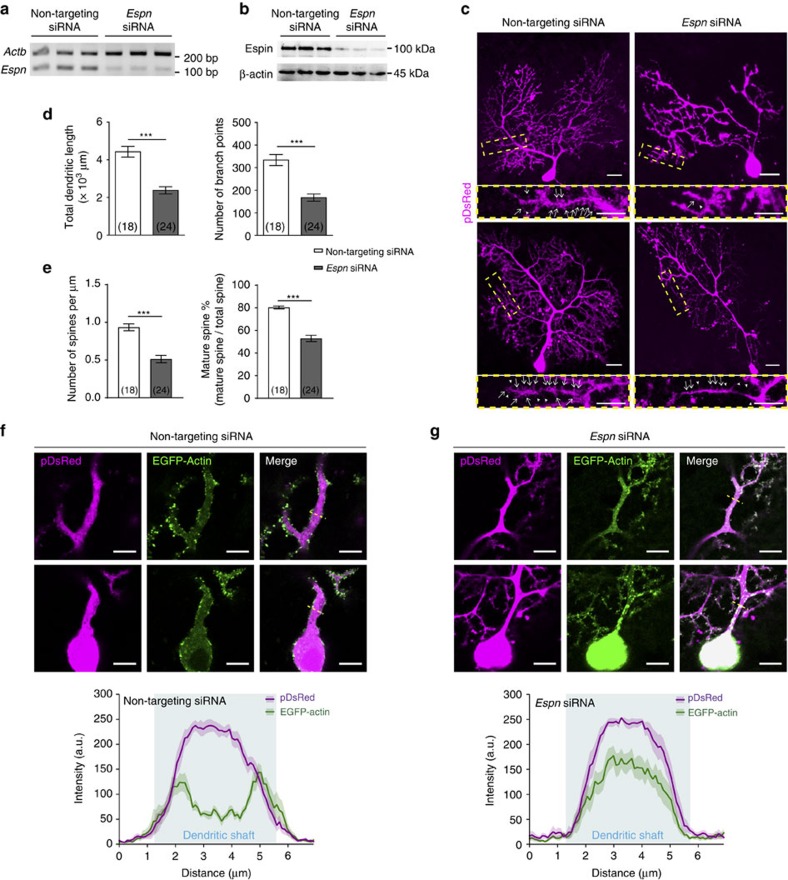
Knockdown of *Espn* disrupts F-actin localization and dendritic development in PCs. (**a**) Semiquantitative RT-PCR showing significant downregulation of *Espn* in cerebellar slice cultures after transfection of *Espn* siRNA; *n*=3 mice per group. (**b**) Western blots showing significant knockdown of Espin protein in stable HEK293A cells expressing Espin-DsRed after transfection with *Espn* siRNA; *n*=3 per group. (**c**) Representative PCs from P14 cerebellum slice cultures co-transfected with pDsRed and nontargeting siRNA (left panels) or *Espn* siRNA (right panels) and collected at 3 days *in vitro* (DIV). Zoom-in images of yellow boxed regions show the dendritic spines of the respective PCs. White arrows point to the mature spines while arrowheads point to the immature spines. Scale bars, 20 μm; 10 μm (zoom-in). (**d**) Bar graphs showing the total dendritic length (left) and the branching number (right) of PCs were significantly reduced after transfection of *Espn* siRNA. (**e**) Bar graphs showing the density of dendritic spines (left) and the percentage of mature spines (right) of PCs were significantly reduced after transfection of *Espn* siRNA. Brackets show the number of transfected PCs analysed. *N*=7–8 mice; *t*-test; ***P<0.001. (**f**,**g**) F-actin cytoskeleton was visualized by co-transfection of EGFP-Actin into P14 PCs transfected with nontargeting siRNA (**f**) or *Espn* siRNA (**g**). Only the central focal planes of the PC proximal dendrites were shown here. Scale bars, 10 μm. Fluorescence intensity profile plots (across the yellow doted lines) reveal the distribution of F-actin in the dendritic shafts of the proximal dendrites of the transfected PCs, respectively. Areas shaded in cyan represent the dendritic shafts. Each profile plot was sampled from 11 different PCs from 4 to 6 different mice and the curves represent the mean values with surrounding shaded regions as s.e.m. Note the shift of F-actin to the centre of the dendritic shafts in the PCs transfected with *Espn* siRNA.

To investigate how *Espn* downregulation affects the dendritic development of PCs, we utilized biolistic transfection to deliver short interfering RNA (siRNA) into PCs in cerebellar slice culture to knock down *Espn ex vivo*. P14 mouse cerebellum was used since it expresses *Espn* robustly (Supplementary Fig. 5a) and also PC dendrites undergo active elongation, branching and spinogenesis at P14 (refs 11, 12). When compared with the controls transfected with nontargeting siRNA, *Espn* siRNA significantly reduced Espin expression in both cerebellum slices and stable HEK293A cells expressing Espin-DsRed (Fig. 4a,b). When Espin was knocked down by the *Espn* siRNA, the distal compartments of PC dendrites, where branching and elongation took place, were severely distorted (Fig. 4c). Quantification of total dendritic length and branching number revealed the significant decreases in dendrite elongation and branching in *Espn* siRNA-transfected PCs as compared with the nontargeting controls (Fig. 4d). Detailed examination of the distal branches of PCs showed that *Espn*-knockdown PCs developed less dendritic spines and many of these remained in the immature filopodium form (that is, elongated spines without enlarged spine heads) (Fig. 4c, zoom-in images). Quantification of dendritic spine number showed that both dendritic spine density and mature spine percentage significantly decreased in *Espn*-knockdown PCs (Fig. 4e). These results demonstrate that Espin is essential for proper dendritic development of PCs.

Since Espin is an actin binding protein that can regulate F-actin organization^31^ (Supplementary Fig. 5b,c), we expected the knockdown of *Espn* would disrupt the F-actin cytoskeleton in PC dendrites. EGFP-Actin was therefore co-transfected with the siRNA to facilitate visualization of F-actin cytoskeleton in the transfected PCs (Fig. 4f,g). Normally, as shown in the nontargeting siRNA-transfected controls, F-actin accumulated in the peripheral regions of dendritic shafts in the proximal dendrites of the PCs (Fig. 4f). However, in *Espn* siRNA-transfected PCs, F-actin was mislocalized and randomly distributed in the central region of dendritic shafts in the proximal dendrites (Fig. 4g). The respective plot profiles across the dendritic shafts clearly revealed the mislocalization of F-actin in the PCs after knockdown of *Espn*. This suggests Espin is responsible for controlling the F-actin localization in PC dendrites.

### F-actin mislocalization in PCs of *Lhx1/5* DKO mutants *in vivo*

**Figure 5:**
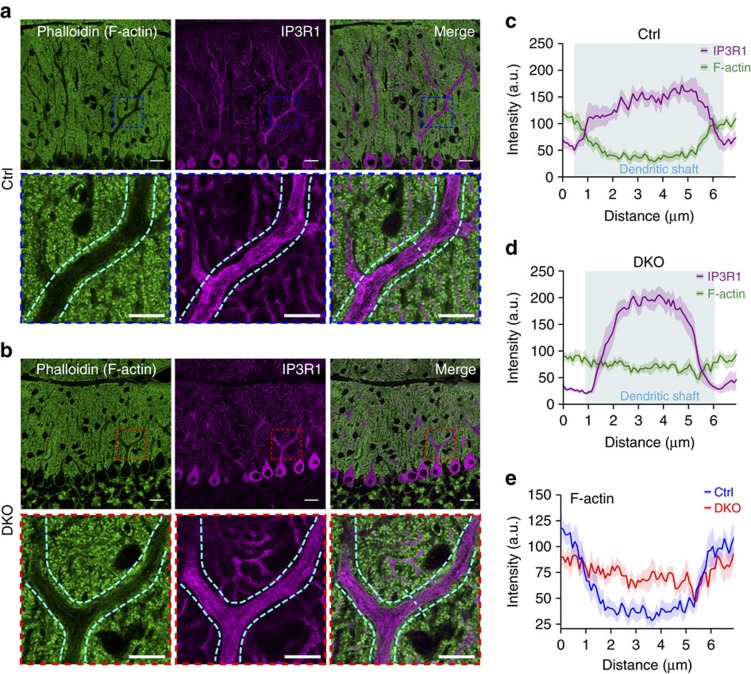
Distorted F-actin localization in the PCs of DKO mutants *in vivo*. (**a**,**b**) Immunostaining of IP3R1 (magenta) and phalloidin staining (green) in the cerebellum sections from the controls (**a**) and the DKO mutants (**b**). Phalloidin (green) stained the F-actin cytoskeleton while IP3R1 (magenta) marked PCs. Lower panels are the zoom-in images of boxed regions respective to the upper panels. Only the central focal planes of the PC proximal dendrites are shown here. Cyan dotted curves highlight the peripheries of the dendritic shafts of the proximal dendrites. Scale bars, 20 μm; 10 μm (zoom-in). (**c**–**e**) Fluorescence intensity profile plots (across the dotted cyan lines) reveal the distribution of F-actin in the dendritic shafts of the proximal dendrites in the PCs from the controls (**c**) and the DKO mutants (**d**). Areas shaded in cyan represent the dendritic shafts. Each profile plot was sampled from 10 different PCs from 4 different mice and the curves represent mean±s.e.m. Note the altered distribution of F-actin in the centre of dendritic shafts in the PCs of the DKO mutants *in vivo* (**e**).

As Espin was downregulated in *Lhx1/5* DKO mutants, the mutant PCs should also encounter the F-actin mislocalization defects *in vivo*. Using phalloidin staining in cerebellum sections (Fig. 5a,b), we found that F-actin was mainly localized in the peripheral regions of dendritic shafts in the proximal dendrites of the control PCs (Fig. 5a). However, in the mutant PCs, F-actin was distributed not only in the peripheral region but also in the central region of dendritic shafts in the proximal dendrites (Fig. 5b). The plot profiles across the dendritic shafts clearly showed the altered F-actin localization in the dendritic shafts of the mutant PCs as compared with the controls (Fig. 5c–e). This shows that the inactivation of Lhx1/5 in PCs leads to F-actin mislocalization in the proximal dendrites that is a phenocopy of the *Espn*-knockdown PCs, suggesting that the *in vivo* defect is mainly caused by Espin downregulation.

### Mutant PCs show spine maturation and synaptogenesis defects

**Figure 6:**
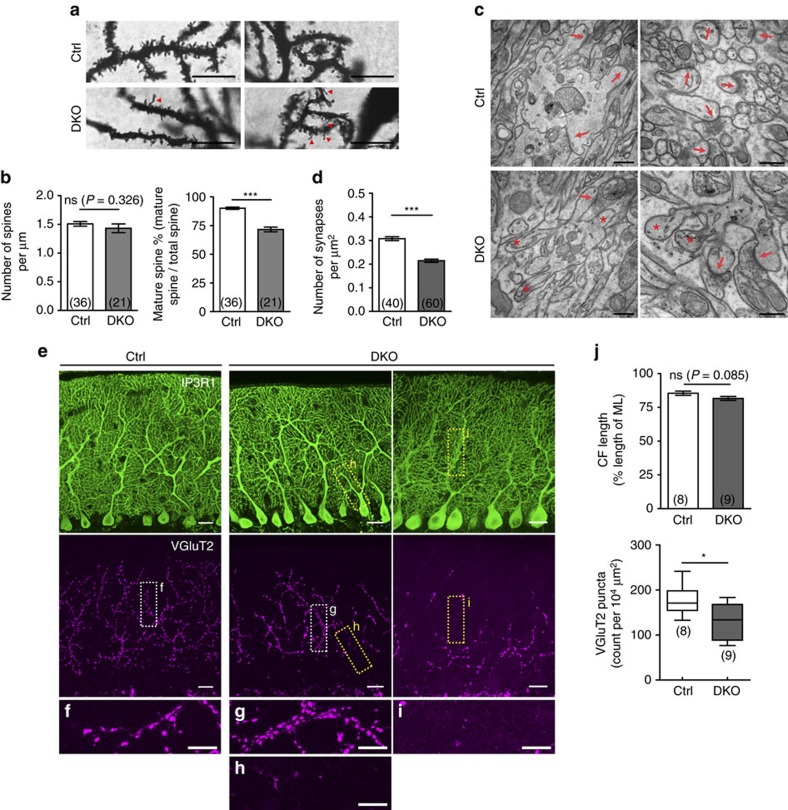
Defects in spine morphogenesis and synaptogenesis in the PCs of DKO mutants *in vivo*. (**a**) High-magnification images of Golgi-impregnated PCs showed the morphology and arrangement of dendritic spines in the controls and the DKO mutants. Arrowheads point to immature filopodium-like dendritic spines. Scale bars, 10 μm. (**b**) Bar graphs showing dendritic spine density of the PCs (left) in the controls and the DKO mutants did not have significant differences but the percentage of mature spines (right) significantly decreased in the PCs of DKO mutant. Brackets show the number of PC dendrites analysed; *N=*3 mice per group. (**c**) Representative electron micrographs from cerebellum of the controls and the DKO mutants. Arrows point the PF-PC synapses and asterisks mark the PC dendritic spines that cannot form synapses. Scale bars, 500 nm. (**d**) Bar graph showing the density of the PF-PC synapses was significantly reduced in the DKO mutants. Brackets show the number of electron micrographs (at 15,000 × magnification) analysed; *N*=2–3 mice per group. (**e**) Immunostaining of IP3R1 (green) and VGluT2 (magenta) showing differences in CF-PC innervations between the controls and the DKO mutants. Zoom-in images of VGluT2 immunostaining (**f**,**g**) showing the normal distribution of CF inputs in the control (**f**) but abnormal clustering of CF inputs on the proximal dendrites of the mutant PCs (**g**). Some proximal dendrites of DKO mutant PCs (**h**,**i**) did not innervate with CFs. Scale bars, 20 μm; 10 μm for (**f**–**i**). (**j**) Bar graphs showing the climbing height of CFs (top) and the density of VGluT2 puncta (bottom) in the cerebella from the controls and the DKO mutants. CF terminals significantly reduced in the mutants. Brackets indicate the number of sections analysed. At least three mice were analysed per group; *t*-test; ns, not significant; *P<0.05 and ***P<0.001.

As mentioned above, dendritic spine morphogenesis of PCs was disrupted when *Espn* was downregulated. We therefore performed Golgi staining to examine if the PC dendritic spines displayed similar defects in *Lhx1/5* DKO mutants *in vivo*. We found that the morphology and the distribution of dendritic spines in the distal dendrites were fairly irregular in the mutant PCs (Fig. 6a). In the mutant PCs, the spines distributed unevenly along the dendrites in which some regions had lots of spines and some regions lacked spines. In addition, more immature filopodia-like dendritic spines were found in the mutant PCs (Fig. 6a, red arrowheads). Statistical analysis showed that, although we found that the overall dendritic spine density was comparable between the mutant PCs and the control PCs, the mature spine percentage significantly decreased by 20.5% in the mutant PCs (Fig. 6b).

Since the majority of PC dendritic spines are responsible for innervation with parallel fibres (PFs) from GCs, we examined whether the defects in spine morphogenesis would affect PF-PC synapses. As revealed by immunostaining of VGluT1 (that is, a presynaptic marker for PF terminals), no observable difference was found between the controls and the mutants (Supplementary Fig. 6a). The percentage area covered by PFs was comparable between the controls and the mutants (Supplementary Fig. 6b). Since the result may imply that PF-PC innervations were unaffected in the mutants or the number of PFs was normal in the mutants, we further examined the PF-PC synapses by transmission electron microscopy (Fig. 6c). In the mutant PCs, although some dendritic spines could still form synapses with PFs (Fig. 6c, red arrows), some did not have postsynaptic density region and failed to form synapses with PFs (Fig. 6c, asterisks). The density of PF-PC synapses therefore decreased by 30.4% in the DKO mutants as compared with the controls (Fig. 6d).

Other than PF-PC innervations, we also studied whether another major excitatory input, climbing fibre (CF), was also affected in the DKO mutants. Immunostaining of VGluT2 (presynaptic marker of CF terminals) showed that the CF-PC innervations were reduced in the DKO mutants as compared with the controls (Fig. 6e). A closer examination revealed that some CF-PC connections were heavily clustered in the proximal dendrites of the mutant PCs (Fig. 6g), whereas no obvious large clusters of CF-PC synapses were observed in the controls (Fig. 6f). At the same time, some mutant PCs lost their connection with CFs, as shown by the absence of VGluT2 signals in their dendrites (Fig. 6h,i). Quantification revealed that the overall CF-PC innervations (that is, VgluT2 puncta count) decreased by 28.3% in the mutants, although the CFs of the mutants could still ‘climb' to the same height as the controls (Fig. 6j). Together, the results provide evidence that Lhx1/5 control dendritic spine morphogenesis and thus synaptogenesis in postnatal PCs.

### Espin controls F-actin localization in PC proximal dendrites

**Figure 7:**
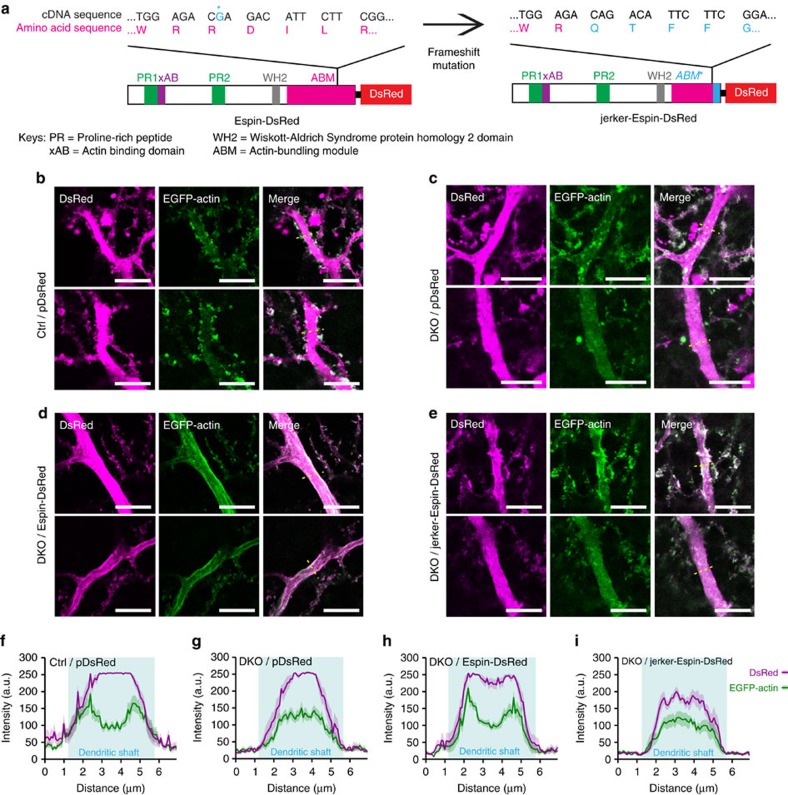
C-terminus of actin-bundling module of Espin is required for proper F-actin localization. (**a**) Schematic representation of Espin-DsRed and jerker-Espin-DsRed. The nucleotide in blue (marked by asterisk) was deleted and resulted in a frameshift mutation in C-terminus of jerker-Espin. The resulting jerker-Espin-DsRed had a shorter and mutated actin-bundling module (ABM*). (**b**–**e**) Central focal planes of the PC proximal dendrites from P14 control (**b**) or DKO mutant (**c**–**e**) co-transfected with EGFP-Actin and pDsRed (**b**,**c**) or Espin-DsRed (**d**) or jerker-Espin-DsRed (**e**). Scale bars, 10 μm. (**f**–**i**) Fluorescence intensity profile plots (across the yellow doted lines) reveal the distribution of F-actin in the dendritic shafts of the proximal dendrites of the transfected PCs, respectively. Areas shaded in cyan represent the dendritic shafts. Each profile plot was sampled from 10 different PCs from 4 to 5 different mice and the curves represent mean±s.e.m. Note that the F-actin localization in the mutant PCs restored to the controls' pattern after transfection of Espin-DsRed.

The defects found in the mutant PCs *in vivo* were all phenocopies of *Espn*-knockdown PCs, implying that the defects in the DKO mutants should be mainly caused by downregulation of Espin. Therefore, we hypothesized that overexpression of Espin in the mutant PCs might rescue the defects observed. Using biolistic transfection, Espin-DsRed was delivered into the PCs in the *ex vivo* slice culture of P14 mutant cerebellum. F-actin localization in the proximal dendrites of transfected PCs was examined through coexpression of EGFP-Actin. Just as with the *in vivo* staining of F-actin, in *ex vivo* cerebellar slice culture the control PCs also had a peripheral distribution of F-actin (Fig. 7b,f), while the mutant PCs still had a random distribution of F-actin in the central regions of the dendritic shafts (Fig. 7c,g). After overexpression of Espin, F-actin of the mutant PCs was restored to the normal position and accumulated in the peripheral regions of the dendritic shafts (Fig. 7d,h), similar to the control PCs. This constitutes direct evidence that Espin is important for F-actin localization in the proximal dendrites of PCs.

**Figure 8:**
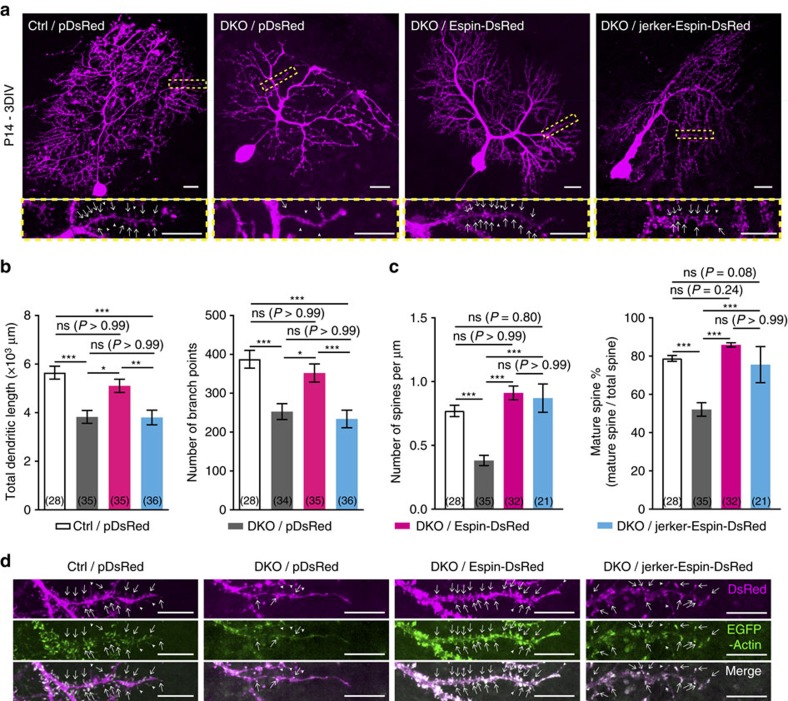
Overexpression of Espin rescues the dendritic defects in the PCs of the DKO mutants *ex vivo*. (**a**) Representative PCs from P14 cerebellum slice cultures of the DKO mutants transfected with pDsRed or Espin-DsRed or jerker-Espin-DsRed and collected at 3 days *in vitro* (DIV). PCs from P14 control mice transfected with pDsRed served as the controls. Zoom-in images of yellow boxed regions show the dendritic spines of the respective PCs. Note that the dendritic trees of the mutant PCs transfected with Espin-DsRed were more extensive and had more mature spines. Scale bars, 20 μm; 10 μm (zoom-in). (**b**) Bar graphs showing the total dendritic length (left) and the branching number (right) of transfected PCs. The dendrite elongation and branching of the mutant PCs restored to the controls' level after transfection of Espin-DsRed. (**c**) Bar graphs showing the density of dendritic spines (left) and the percentage of mature spines (right). Spinogenesis and spine maturation of the mutant PCs significantly increased after transfection of Espin-DsRed or jerker-Espin-DsRed. Brackets show the number of transfected PCs analysed. *N*=4–9 mice. Kruskal–Wallis test; ns, not significant; *P<0.05, **P<0.01 and ***P<0.001. (**d**) Distal dendrites of PCs from the controls or the mutants co-transfected with EGFP-Actin and pDsRed or Espin-DsRed or jerker-Espin-DsRed. White arrows point to the mature spines while arrowheads point to the immature spines. Both normal Espin and jerker-Espin were highly colocalized with F-actin in the dendritic spines of the transfected PCs. Scale bars, 10 μm.

To further investigate how Espin regulates F-actin localization, we generated a mutated Espin-DsRed construct (that is, jerker-Espin-DsRed) carrying the *jerker* mutation^32^,^33^, a spontaneous frameshift mutation in the coding sequence of the actin-bundling module (ABM) (Fig. 7a). The *jerker* mutation created a nucleolar localization sequence in the C-terminus of ABM^31^,^33^. Consistent with previous studies^31^,^33^, jerker-Espin-DsRed was localized and bundled F-actin only in the nuclei of the transfected cells (Supplementary Fig. 7). Unexpectedly, jerker-Espin ‘spread' to the dendrites, and was not restricted to the nuclei when transfected into P14 mutant PCs (Figs 7e and 8a). When we examined F-actin localization in the proximal dendrites of the mutant PCs transfected with jerker-Espin-DsRed, we found that F-actin was still mislocalized and randomly distributed in the centre of the dendritic shafts (Fig. 7e,i). This suggests that the C-terminus of ABM of Espin is required for proper F-actin localization in the proximal dendrites of the PCs.

### Overexpression of Espin rescues the defects in DKO mutants

Apart from F-actin localization, we also examined whether the dendritic defects in the DKO mutants could be rescued by overexpression of Espin. Compared with the mutant PCs transfected with pDsRed, mutant PCs transfected with Espin-DsRed had more extensive dendritic arborization with more mature dendritic spines (white arrows) (Fig. 8a). Quantification showed that the total dendritic length and branching number of the mutant PCs were restored to the control levels after overexpression of Espin (Fig. 8b). Spine density and mature spine percentage of the DKO mutants also returned to the control level after transfection of Espin-DsRed (Fig. 8c). On the other hand, overexpression of mutant Espin (that is, jerker-Espin-DsRed) did not increase the total dendritic length and branching number, which were significantly lower than the mutant PCs transfected with Espin-DsRed and the control PCs (Fig. 8b). However, transfection of jerker-Espin surprisingly led to a significant increase in the number of dendritic spines, particularly mature spines, in the mutant PCs (Fig. 8c). Examination of the transfected mutant PCs revealed that jerker-Espin, like normal Espin, could still bind and bundle F-actin in the dendritic spines (white arrows and arrowheads) in the distal dendrites (Fig. 8d). This might explain why the mutant PCs transfected with jerker-Espin-DsRed were comparable to the control PCs and the mutant PCs transfected with Espin-DsRed, in terms of spine density and mature spine percentage (Fig. 8c). The phenotypic rescue by Espin overexpression implies that Espin downregulation is the major cause of the observed defects in *Lhx1/5* DKO mutants.

### Mutant PCs show aberrant electrophysiological properties

**Figure 9:**
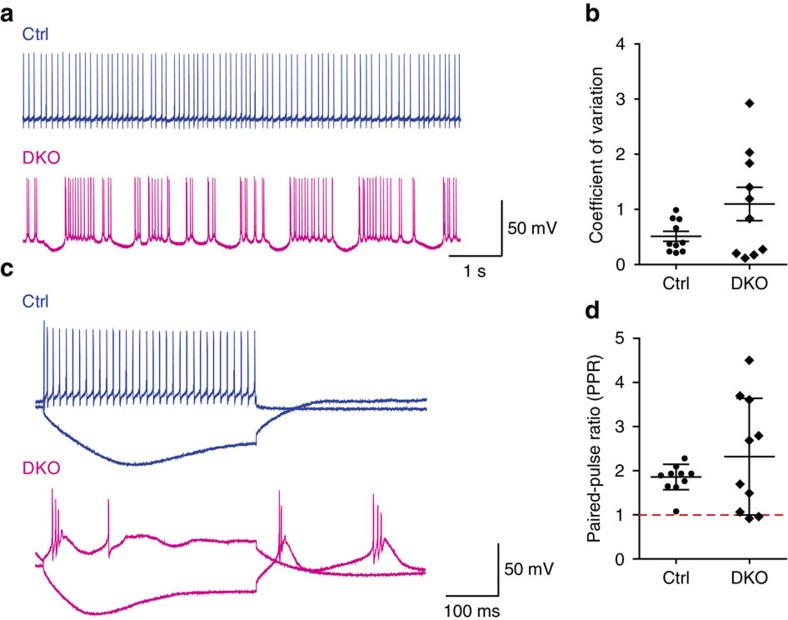
Aberrant electrophysiological properties in the PCs of *Lhx1/5* DKO mutants. (**a**) Typical traces of PC spontaneous firing showing differences in the firing patterns of spontaneous spike between the control PCs (top) and the mutant PCs (bottom). (**b**) Scatter plot showing the coefficient of variation (CV) of interspike intervals of the PCs from the controls or the DKO mutants. (**c**) Representative traces of PC firing in response to depolarizing and hyperpolarizing current injections. Regular repetitive firing and hyperpolarization with inward rectification are shown by the control PCs (top) but bursts of action potentials reminiscent of complex spikes during depolarization and at the termination of a hyperpolarizing response are shown in the mutant PCs (bottom). (**d**) Scatter plot showing the difference in paired-pulse ratio (PPR) between the control PCs and the mutant PCs after delivery of dual stimuli to PFs. Note the greater variability in PPRs in the PCs of the DKO mutants. Data points above the dotted line represent paired-pulse facilitation, while data points below the dotted line represent paired-pulse depression. For all scatter plots, the bars indicate the mean values and 10 PCs from 5 mice were analysed for each group.

Since abnormal PF-PC and CF-PC innervations may affect the electrophysiology of PCs, we examined the electrophysiological properties of the DKO mutant PCs. From the recordings of spontaneous PC firings, we found that the control PCs characteristically exhibited regular firings (Fig. 9a, upper panel) but the mutant PCs had a strong tendency to generate irregular spiking (Fig. 9a, lower panel). We quantified the regularity of the firing of the two groups of PCs by determining the coefficient of variation (CV) of inter-spike intervals. As shown in Fig. 9b, high values of CV were found only in some mutant PCs. The CV of the mutant group ranged from 0.12 to 2.92, while that of the control group ranged from 0.22 to 0.99.

In response to depolarizing and hyperpolarizing current injections, the control PCs usually displayed regular repetitive firing and hyperpolarization with inward rectification, respectively (Fig. 9c, upper panel). In contrast, the mutant PCs easily generated bursts of action potentials reminiscent of complex spikes that were also commonly observed at the termination of a hyperpolarizing response (Fig. 9c, lower panel). Since complex spikes are triggered by CF excitation^34^, the appearance of complex spike-like firings upon current injections may be a consequence of altered CF-PC innervation patterns in the mutant PCs.

When we analysed the synaptic currents evoked by rapidly stimulating the PFs twice, the mutant PCs displayed an unusual wide range of paired-pulse ratio (PPR, ratio of the amplitude of the second excitatory postsynaptic current (EPSC) to the first EPSC) (Fig. 9d). In the control PCs, paired-pulse facilitation centred around 1.8 with relatively little variation. However, in the mutant PCs, very big PPRs were frequently encountered. In addition, paired-pulse depression, which was atypical of the PF-PC synapse, was detected in some mutant PCs. This may be related to reduced PF-PC synapses in the mutants as described above. After deletion of *Lhx1/5*, the postsynaptic functions of the PCs may have been affected resulting in aberrant electrophysiological properties.

## Discussion

**Figure 10:**
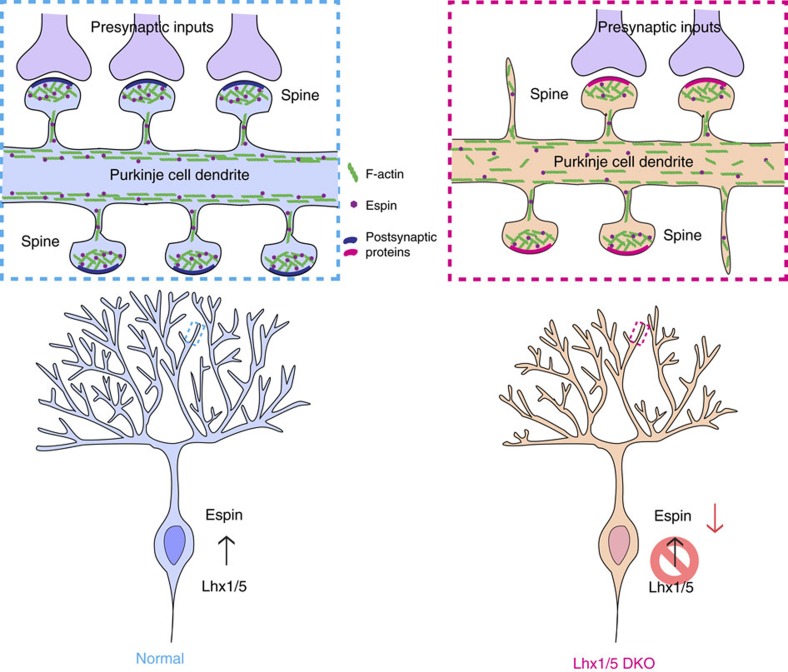
Summary illustration of the role of Lhx1/5 in PC dendritogenesis and spine morphogenesis. In normal mouse cerebellum, Lhx1/5 activate the expression of Espin that regulates the organization of F-actin in PC dendrites and thereby controls dendritogenesis and spine morphogenesis of PCs. The dendritic spines can maturate normally and form synapses with the presynaptic inputs. In *Lhx1/5* DKO mutant cerebellum, Espin expression is downregulated due to inactivation of *Lhx1/5*. As a result, less Espin can be found in PC dendrites and thus F-actin becomes disorganized in PC dendrites. This disrupts dendritogenesis and the maturation of dendritic spines and results in loss of synapses with some presynaptic inputs.

In this study, we provide genetic evidence that the LIM-homeodomain transcription factors Lhx1 and Lhx5 govern dendritogenesis and dendritic spine morphogenesis in postnatal PCs through regulating Espin expression. Conditional knockout of Lhx1/5 in postnatal PCs leads to downregulation of Espin, resulting in F-actin mislocalization in the proximal dendrites of PCs. This thereby impairs the elongation and branching of PC dendrites in *Lhx1/5* DKO mutants. Since spine maturation is also disrupted due to reduced Espin expression, synaptogenesis and electrophysiological properties of the mutant PCs become abnormal, causing ataxia in the DKO mutants (Fig. 10).

Lhx1 and Lhx5 belong to the LIN-11 group of LIM-homeodomain protein family and act as transcriptional activators^29^. Previous studies have shown that Lhx1/5 are functional redundant in the early differentiation of PCs in the embryonic cerebellum^9^ and in the maintenance of inhibitory interneurons in the dorsal horn of the spinal cord^35^,^36^. This redundancy was also found here in regulating the dendritic development of postnatal PCs, indicating that Lhx1 and Lhx5 share common transcriptional regulatory targets. To identify these targets, we used a microarray analysis to compare the gene expression profiles of the mutant cerebella with the controls. In this study, we focused on one of the downregulated genes, *Espn,* whose expression is significantly reduced in the mutant PCs. Due to the redundant role of Lhx1/5, either Lhx1 or Lhx5 alone are sufficient to activate *Espn* expression as shown in the SEAP assay. This explains why the control mice with one functional copy of either *Lhx1* or *Lhx5* have normal phenotypes. Our results also suggest that *Espn* is a common regulatory target of Lhx1/5 in postnatal PCs.

We predicted that *Espn* plays a more important role in dendrite elongation and branching and spinogenesis since *Espn* starts to be expressed in the cerebellum from P9, corresponding to the later phase of dendritic development in PCs^11^,^12^. To determine the functional role of Espin in PCs, we specifically knocked down *Espn* in wild-type PCs by siRNA. The resulting phenotypes of *Espn*-knockdown PCs resemble that in the DKO mutant PCs, in which both show disrupted dendritogenesis and spinogenesis in the *ex vivo* cultures. This suggests that the dendritic defects shown in the PCs of *Lhx1/5* DKO mutants are mainly caused by downregulation of Espin. The decrease in dendritic spine density was only observed in the *ex vivo* cultures but not in the *in vivo* Golgi staining. This may be due to the differences between the *ex vivo* culture and the *in vivo* environments. Since *ex vivo* cultures involve slicing the cerebellum, some presynaptic inputs of PCs and thus parts of the extrinsic stimuli for spinogenesis will be disrupted. The mutant PCs may be more susceptible to the environmental changes, thus having a more drastic phenotype in *ex vivo* environments.

Since we proposed that the dendritic defects in *Lhx1/5* DKO mutants were mainly caused by Espin downregulation, we speculated that Espin overexpression would rescue the defects in the mutant PCs. As expected, Espin overexpression not only increases dendritogenesis and spinogenesis in the mutant PCs, but also restores them to a level that is comparable to the control PCs. F-actin localization in the mutant PCs also returns to a normal status, with a distribution profile resembling that of the control PCs. This rescue experiment provides strong evidence in favour of Espin being a major downstream effector of Lhx1/5 for controlling dendritic development in PCs.

Espin is an actin regulatory protein^31^,^33^ that can control the formation and elongation of protrusion structures in different cell types by regulating F-actin cytoskeleton organization^32^,^37^,^38^,^39^. To determine how Espin regulates F-actin localization in PCs, Espin-DsRed carrying the *jerker* mutation^32^ was transfected into the mutant PCs. We expected that jerker-Espin would only be expressed inside the cell nucleus because the *jerker* mutation introduces a nucleolar localization sequence in the C-terminus of ABM^31^,^33^. However, jerker-Espin was also expressed in the dendrites of the transfected PCs. Since jerker-Espin has functional actin binding and bundling domain^31^, it can still bind and bundle F-actin in the PC dendrites. However, in contrast to normal Espin, jerker-Espin cannot restore F-actin localization in the proximal dendrites of the mutant PCs. Besides, dendritogenesis of the mutant PCs is still compromised after overexpression of jerker-Espin. This implies that the C-terminus of ABM of Espin is responsible for proper F-actin localization in the proximal dendrites of PCs that in turn is required for dendritogenesis.

On the other hand, jerker-Espin is highly colocalized with F-actin in the dendritic spines of the transfected PCs, indicating a normal F-actin distribution in the distal dendrites. This suggests that F-actin localization in the proximal dendrites and in the distal dendrites may be regulated by different mechanisms. Previously, Sekerková *et al*.^28^ found that Espin can interact with IRSp53 that has been shown to regulate dendritic spine morphogenesis in hippocampal neurons^40^. IRSp53 is associated with postsynaptic density (PSD) in the PC dendritic spines^41^. It has been found that the SH3 domain of IRSp53 not only interacts with PSD but also proline-rich (PR) domains of actin-regulatory proteins including Espin^42^. Therefore, Espin may associate with PSD by interacting with IRSp53 in the dendritic spines. Since the PR domains remain unaffected in jerker-Espin, jerker-Espin should be able to interact with IRSp53 and thus localize in the dendritic spines to organize F-actin cytoskeleton in the distal dendrites of PCs. As formation of the F-actin network and F-actin bundles promotes spinogenesis and spine maturation^43^, jerker-Espin could increase the density of dendritic spines, especially mature spines in the mutant PCs. These data illustrate the importance of normal F-actin distribution in promoting the dendritic development of PCs.

Indeed, the spontaneous Espin mutants, *jerker* mice, did not show obvious morphological changes in PCs in a gross histological study^44^. The j*erker* mutants suffer from deafness, imbalance and shaker–waltzer behaviour and these phenotypes have been reported to be caused by hair cell loss in the inner ear of *jerker* mice^32^. Due to the striking phenotypes in the inner ears, previous studies on Espin have focused on its role in inner ear development, but not in PC dendritic development. Since there has been no in-depth examination of PCs in the *jerker* mutants, it remains unclear whether *jerker* mutants also share the same defects as our DKO mutants in PC dendritic development. Unlike the *ex vivo* experiments we performed in this study, previous studies suggest jerker-Espin is unstable and cannot exist under *in vivo* conditions^31^,^32^,^33^. However, since those studies did not mention the expression of jerker-Espin in cerebellum, we cannot exclude the possibility that jerker-Espin may be expressed in the PCs. If jerker-Espin were expressed in the dendrites of PCs *in vivo*, the PCs of *jerker* mice would differ from our mutant PCs based on the findings presented here. Nevertheless, we speculate that the PCs of *jerker* mutants would still encounter problems at least in terms of dendrite elongation and branching.

Our results illustrate the similar roles of Espin in various cell types in regulating the formation of different membrane protrusions, implying that the molecular mechanism underlying the formation of filopodia or similar structures may involve common factors. In the future, it will be worth investigating what additional factors interact with this common factor to facilitate the differentiation of membrane protrusions for specific functions.

Apart from the spine morphogenesis defects, the mutant PCs also encounter problems in synaptogenesis with PFs and CFs, thus leading to the aberrant electrophysiological properties observed. Our electrophysiological analysis reveals that the mutant PCs give bursts of complex spike-like firings upon depolarizing and hyperpolarizing current injections. A previous study has suggested that complex spike-like firings are evoked only when high intensity depolarizing currents are injected into PCs^45^. However, here we injected low-intensity currents, and hence it is abnormal for the mutant PCs to give bursts of complex spike-like firings. Since complex spike-like bursts are triggered by activation of Ca^2+^-Na^+^ bursts instead of simple Na^+^ spikes^45^, calcium signalling of the mutant PCs may have defects. One of the possible defects would be the mislocalization of calcium channels due to clustering of CF-PC synapses in the proximal dendrites. Mislocalization of calcium channels may make the channels easier to open upon stimulation. This will require further studies to confirm whether calcium signalling is perturbed upon *Lhx1/5* inactivation. On the other hand, the mutant PCs show greater variability in the PPR than the control PCs. This is probably caused by a defect in the maintenance of PF-PC synapses. PCs may become overexcited or underexcited upon PF stimulation. Another possible cause would be the mislocalization of glutamate receptors due to F-actin mislocalization, but this needs to be verified.

From the microarray analysis, we can identify other downregulated factors that may also contribute to the abnormal dendritic development in the mutant PCs. For example, *Itpka* has been reported to regulate dendritic spine morphogenesis and shape synaptic Ca^2+^ transients in developing hippocampal neurons^46^, while *Fgf7* controls the differentiation of inhibitory synapses in CA3 pyramidal neurons^47^. Although downregulation of these factors may also contribute to the observed defects, phenotypic rescue by Espin overexpression suggests that Espin downregulation is one of the major causes of abnormal dendritic development in *Lhx1/5* DKO mutants. Our work presents points to a novel mechanism as to how dendritic development of PCs is regulated by innate genetic factors in the postnatal cerebellum.

## Methods

### Mice

The generation of *Lhx5 fx* mice is described in the Supplementary Methods. All the mice were maintained in C57BL6 background. We crossed *Pcp2*-*Cre*^22^ mice with *Lhx1 fx*^19^ mice and *Lhx5 fx* mice to obtain *Pcp2*-*Cre*/+; *Lhx1*^*fx/fx*^; *Lhx5*^*fx/fx*^ (DKO) mutant progeny. Either *Pcp2*-*Cre*/+; *Lhx1*^*fx/+*^; *Lhx5*^*fx/fx*^ or *Pcp2*-*Cre*/+; *Lhx1*^*fx/fx*^; *Lhx5*^*fx/+*^ mice served as controls. All mice were kept in the Animal House of the Chinese University of Hong Kong with a 10:14 h light/dark cycle. All animal procedures were conducted with the approval of the Animal Experimentation Ethics Committee of The Chinese University of Hong Kong (Ref No.: 07/042/ERG). Mice at 1–6 months of age of either sex were used for experiments unless specifically indicated.

### Motor behavioural analysis

For rotarod test, mice were subjected to a 2-day training scheme before measurement. On days 1 and 3, mice were trained on the rotarod (Panlab) at 0 r.p.m. for 1 min, and then at 10 r.p.m. for 1 min. On day 4, mice were placed on the rotarod with increasing speed (accelerating) from 4 to 40 r.p.m. in 2 or 5 min, and with fixed speed (stationary) at 15 r.p.m. for a maximum of 5 min. Five measurements were taken for individual mouse in each test with 5 min rest between each measurement. The mean values were used for statistical analysis.

For elevated balance beam test, mice were trained on days 1 and 3 to walk along an elevated and inclined beam 2 cm wide and 70 cm long^23^. On day 4, the time to complete the 70 cm distance and number of slips were recorded. Five measurements were taken for each mouse with 1 min rest between each measurement. The mean values were used for statistical analysis.

For footprint analysis, the forepaws and hindpaws of mice were painted with nontoxic dye of different colours. Mice were then allowed to run through a cardboard tunnel (10 cm × 10 cm × 85 cm) lined with white paper. Stride length, forelimb–hindlimb overlap for five consecutive steps were measured. Median values were used for statistical analysis.

### Gene expression microarray analysis

Total cerebellum RNA from 12-week-old male controls (*Pcp2*-Cre/+; *Lhx1*^*fx/+*^; *Lhx5*^*fx/fx*^) and DKO mutants (*n*=3 mice for each genotype) was subjected to gene expression profiling using an Agilent microarray platform (Agilent Technologies). Detailed experimental procedures are described in the Supplementary Methods. Significantly expressed genes (*P*<0.05) with at least fivefold differential regulation are listed in Supplementary Tables 1 and 2.

### Quantitative RT-PCR

Total cerebellum RNA from 3-week-old controls and DKO mutants was extracted by TRIzol reagent (Life Technologies). The RNA was then reverse-transcribed to complementary DNA (cDNA) using M-MLV (Moloney murine leukaemia virus) Reverse Transcriptase (Life Technologies) with oligo(dT)_20_ primers. For real-time quantitative reverse transcription PCR (RT-PCR), diluted cDNA samples were amplified by QuantiFast SYBR Green PCR kit (Qiagen) using specific primers with PCR profiles of 95 °C (5 min) followed by 40 cycles of 95 °C (15 s), 62 °C (20 s) and 72 °C (20 s). Fluorescence was measured by Bio-Rad CFX96 Real-Time PCR Detection System at the end of each cycle. Each individual sample was assayed in duplicate and gene expression was normalized with *Gapdh* expression.

Semiquantitative RT-PCR was performed using i-*Taq* DNA polymerase (Intron Biotechnology) with PCR profile: 94 °C (2 min), 30 cycles of 94 °C (20 s), 65 °C (20 s) and 72 °C (20 s), followed by a final extension at 72 °C for 5 min. PCR products were separated by 1.8% agarose gel electrophoresis and visualized by Bio-Rad Gel Doc XR. Gene expression was normalized with *β-actin* expression. Primer sequences for RT-PCR are listed in Supplementary Table 3.

### DNA constructs and recombinant proteins

For promoter assays, the sequence of the putative promoter of *Espn* was obtained from the Eukaryotic Promoter Database^48^. The promoter DNA was amplified from C57BL6 genomic DNA and cloned into pSEAP2-Enhancer vector (Clontech) to construct a Espn-pm-SEAP plasmid. Espn-pm-SEAP was mutated by PCR to generate a mutation in the potential binding site of Lhx1/5 homeodomain^30^ (Espn-pm-mut-SEAP).

FL and truncated (either LIM domain (LIM) or homeodomain (Hom) only) Lhx1/Lhx5 expression constructs (FLAG-Lhx1/5) were obtained by subcloning the respective cDNA into pCMV-Tag2A (Stratagene). DsRed-tagged Espin FL (Espin-DsRed) construct was generated by subcloning *Espn* cDNA into a pDsRed-monomer-N1 vector (Clontech). For visualization of F-actin in cerebellar slice cultures, EGFP-tagged actin (EGFP-actin) construct was generated by subcloning *Actb* cDNA into pEGFP-C1 (Clontech). An Espin expression construct carrying the *jerker* mutation^32^ (jerker-Espin-DsRed) was obtained by introducing the corresponding frameshift mutation to the Espin-DsRed construct by PCR. A fragment of *Espn* cDNA was subcloned into pBluescript II KS- (Stratagene) to generate Espn-pBluescript for *in vitro* synthesis of *Espn* riboprobes for *in situ* hybridization.

All cDNAs for the generation of DNA constructs were amplified from the total cDNA of C57BL6 mouse cerebellum by KAPA HiFi DNA Polymerase (Kapa Biosystems) with the primers listed in Supplementary Table 3.

### Western blotting

Dissected cerebellum was lysed with SDS lysis buffer (1% SDS, 10% glycerol, 5% β-mercaptoethanol and 125 mM Tris-HCl, pH 6.8). The tissue was homogenized and then boiled at 100 °C for 10 min. To extract proteins from HEK293A cells, the cells were lysed with NP-40 lysis buffer (1% NP-40, 0.5% sodium deoxycholate, 150 mM NaCl and 50 mM Tris-HCl, pH 7.5, with Complete protease inhibitor cocktails (Roche)). All lysates were centrifuged and the supernatant were collected for separation by 10% SDS–polyacrylamide gel electrophoresis. The separated proteins were blotted to polyvinylidene difluoride membrane by Bio-Rad Trans-Blot Turbo Transfer System. The membrane was blocked with 5% non-fat dry milk in Tris-buffered saline with 0.1% Tween-20 for 1 h at room temperature. The blocked membrane was incubated with 1:1,000 mouse anti-Espin (BD Biosciences, 611656) or 1:1,000 rabbit anti-β-actin (Cell Signaling, 4967S) or 1:1,000 rabbit anti-FLAG (Sigma, F7425) diluted in 2% ECL Prime Blocking Reagent (GE Healthcare) at 4 °C overnight. After washing, the membrane was incubated with respective secondary antibodies conjugated to horseradish peroxidase (HRP) (GE Healthcare) for 1 h at room temperature. The signal was detected using ECL Western Detection Reagent (GE Healthcare) or SuperSignal West Femto Maximum Sensitivity Substrate (Thermo Scientific).

### Section *in situ* hybridization

Cerebella were dissected from the adult control and DKO mice and snap-frozen in OCT compound (Tissue-tek). Then, 15 μm sections were cut using Leica CM1950 cryostat and briefly fixed with 4% paraformaldehyde (PFA) in diethyl pyrocarbonate-treated phosphate-buffered saline (PBS) for 10 min. After prehybridization treatment, the sections were hybridized with the digoxigenin-labelled riboprobes against *Espn*, synthesized using transcriptional kit (Roche) from Espn-pBluescript with T3 RNA polymerase (Promega), at 65 °C overnight. After several rounds of washing with different dilutions of SSC, the sections were blocked at room temperature for 1 h. The sections were then incubated with 1:1,000 anti-digoxigenin alkaline phosphatase-conjugate (Roche) at 4 °C overnight. The signals were visualized by staining with 250 μg ml^−1^ nitroblue tetrazolium and 125 μg ml^−1^ BCIP (5-bromo-4-chloro-3-indolyl-phosphate) at 37 °C overnight. Images of sections were captured by Olympus BX43 microscope equipped with DP72 camera.

### SEAP gene reporter assay

To evaluate the effect of Lhx1/5 on *Espn* promoter activity, FLAG-Lhx1/5-FL constructs were co-transfected with the Espn-pm-SEAP plasmid into HEK293A cells using jetPRIME reagent (Polyplus Transfection). Empty expression vector (pCMV-Tag2A) and truncated Lhx1/5 expression constructs (FLAG-Lhx1/5-LIM/Hom) served as controls. The HEK293A cells were maintained in Dulbecco's modified Eagle's medium supplemented with 10% fetal bovine serum FBS, 1 × GlutaMAX and 1 × penicillin–streptomycin (Life Technologies) at 37 °C humidified 5% CO_2_ incubator. At 2 days after transfection, the culture medium was collected and assayed by Phospha-Light SEAP Reporter Gene Assay System (Life Technologies). The chemiluminescent signal was measured by Lumi-Imager F1 (Roche). Uncropped blots and gels are provided in Supplementary Fig. 8.

### *In vivo* chromatin immunoprecipitation

*In vivo* chromatin immunoprecipitation of Lhx1/5 in mouse cerebellum was performed according to the previously described protocols^49^,^50^ with modifications. Cerebella from 3-week-old controls and DKO mutants were finely minced into small pieces in PBS. The samples were incubated with 1:10 volume of crosslinking buffer (11% PFA, 0.1 M NaCl, 1 mM EDTA, 0.5 mM EGTA and 50 mM HEPES, pH 7.6) for 10 min with agitation at room temperature. PFA fixation was quenched by incubating with 125 mM glycine for 5 min. Crosslinked samples were washed twice with PBS and lysed with Lysis Buffer 1 (140 mM NaCl, 1 mM EDTA, 10% glycerol, 0.5% NP-40, 0.25% Triton X-100 and 50 mM HEPES, pH 7.6, with Complete Mini protease inhibitors) and Lysis Buffer 2 (200 mM NaCl, 1 mM EDTA, 0.5 mM EGTA and 10 mM Tris, pH 7.6, with Complete Mini protease inhibitors) sequentially for 10 min each. The samples were centrifuged at 2,000 *g* for 10 min at 4 °C before changing lysis buffer. After lysis, the pellets were resuspended with Sonication Buffer (1 mM EDTA, 0.5 mM EGTA, 1% Triton-X100, 1% sodium deoxycholate and 10 mM Tris-HCl, pH 7.6, with Complete Mini protease inhibitors) and sonicated with 20 pulses of 1 s pulse for 15 rounds at 50% power (Branson Sonifier 150) on ice with 1 min rest between each round. The chromatin solution was centrifuged at 10,000 *g* for 10 min at 4 °C and the supernatant was precleared with Protein G Agarose (Santa Cruz) at 4 °C overnight. The precleared chromatin was then incubated with 10 μg rabbit anti-LIM1 antibody (Abcam ab14554) at 4 °C overnight. Chromatin without incubation with any antibodies served as negative control (Mock). After antibody incubation, the chromatin was immunoprecipitated with Dynabeads Protein G (Life Technologies) at 4 °C for 1–2 h. The beads were collected and washed five times with RIPA buffer (1 mM EDTA, 0.7% deoxycholic acid, 1% NP-40, 0.5 M LiCl and 50 mM HEPES, pH 7.6) and once with TE buffer (10 mM Tris-HCl, pH 8.0, and 0.1 mM EDTA). Chromatin was eluted with Elution Buffer (50 mM Tris, pH 8.0, 10 mM EDTA and 1% SDS) at 65 °C for 10 min. The eluted chromatin was reversely crosslinked with Elution Buffer at 65 °C overnight and then treated with Proteinase K at 55 °C for 1–2 h. The chromatin was extracted and purified by phenol/chloroform. The purified DNA was subjected to semiquantitative PCR with primers listed in Supplementary Table 3 to test the presence of the *Espn* promoter in the immunoprecipitated chromatin.

### Knockdown of *Espn* by siRNA

ON-TARGETplus SMARTpool siRNA for *Espn* (target sequences: 5′-GAGAGGGAGCAGAAGCGAA-3′; 5′-GACGAGACAUUCUUCGGAA-3′; 5′-CGAAAGAACAGUCGGAGAA-3′; 5′-GGACACUAGGCUACGACGA-3′) (Dharmacon, L-065563-01-0005) was used to knock down *Espn*. To evaluate the knockdown efficiency of the siRNA, the siRNA was transfected by INTERFERin (Polyplus Transfection) into stable HEK293A cells expressing Espin-DsRed following the manufacturer's instructions. ON-TARGETplus SMARTpool nontargeting siRNA (Dharmacon, D-00180-10-05) was used as the control. The final working concentration of the siRNA was 10 nM. The transfected cells were maintained in the HEK293A culture medium supplemented with 500 μg ml^−1^ G418 (Gibco) at 37 °C in 5% CO_2_ incubator for 2 days and then collected for western blot analysis.

### Organotypic culture of cerebellar slices

Cerebella from P14 mice were dissected and freshly sectioned into 250 μm thick slices in ice-cold PBS with 0.6% glucose using Leica VT1000S vibratome. The cerebellar slices were then washed with cerebellar slice culture medium (Neurobasal-A medium, 1 × B-27 supplement, 1 × GlutaMAX and 1 × penicillin–streptomycin, Life Technologies) twice and placed on 30 mm cell culture inserts (0.4 μm pore size, Millipore). The culture inserts were then placed into 6-well plate with 1 ml culture medium per well and maintained in 37 °C humidified 5% CO_2_ incubator for 3–4 h before biolistic transfection.

### Biolistic transfection

To transfect single PCs in cerebellar slice cultures, siRNA/DNA- or DNA-coated gold particles were delivered into the cells using Helios Gene Gun (Bio-Rad). For *Espn* knockdown experiments, siRNA was co-transfected with pDsRed-monomer-N1 and EGFP-Actin plasmids to facilitate the visualization of transfected PCs and their F-actin cytoskeleton. Briefly, 10 mg of 1 μm gold particles (Bio-Rad) were coated with 50 μM spermidine followed by precipitation of 1 μg *Espn* siRNA, 5 μg pDsRed-monomer-N1 and 5 μg EGFP-Actin plasmids to the gold particles with 1 M CaCl_2_. Nontargeting siRNA was used as the control for the knockdown experiments. Similarly, for overexpression of Espin, 10 mg of 1.6 μm gold particles (Bio-Rad) were coated with 5 μg Espin-DsRed and 5 μg EGFP-Actin plasmids. pDsRed-monomer-N1 was used as the control for the overexpression experiment. The siRNA/DNA- or DNA-coated gold particles were washed three times with absolute ethanol and coated into Tefzel tubing (Bio-Rad) that was trimmed into small cartridges for bombardment. The siRNA/DNA or DNA was bombarded into the cerebellar slices with two helium pressure pulses at 180 psi. The transfected cerebellar slices were maintained at 37 °C in 5% CO_2_ incubator for 2 days. The slices were then fixed with 4% PFA in PBS for 15 min at room temperature. Confocal *z*-stack images (step size=0.5 μm) of transfected PCs were captured using Leica TCS SP8 with 63 × water lens for the analysis of F-actin localization and spine density.

### Histological analysis

Dissected cerebella from adult mice (1–6 months old) were fixed in 4% PFA in PBS at 4 °C for 2–16 h. For preparation of paraffin sections for haematoxylin and eosin staining or immunohistochemistry, fixed cerebella were dehydrated, paraffin embedded and sectioned into 5 μm thick sections with a microtome. For preparation of floating sections for immunofluorescence, fixed cerebella were sectioned into 50 μm thick floating sections in ice-cold PBS with a vibratome. For immunohistochemistry, antigen retrieval was performed by microwave treatment in 10 mM sodium citrate buffer (pH 6.0) if needed. The sections were blocked with blocking solution (0.2% bovine serum albumin, 10% lamb serum and 0.1% Triton X-100 in PBS) at room temperature for 1 h or at 4 °C overnight. The blocked sections were then incubated with primary antibodies at 4 °C for 1–2 overnight. The primary antibodies and the dilutions used for immunostaining are listed in Supplementary Table 4. After washing, the sections were incubated with respective secondary antibodies at room temperature for 1 h or overnight at 4 °C. Secondary antibodies were conjugated with HRP (Millipore, 1:250) or Alexa Fluor 488/568 (Life Technologies, 1:1,000). F-actin staining was performed with 1:1,000 Alexa Fluor 488 phalloidin (Life Technologies) at 4 °C overnight if needed. For immunohistochemistry, HRP signals were visualized by incubating the sections with diaminobenzidine (Dako) and counterstained with haematoxylin. Images of haematoxylin and eosin and immunohistochemistry sections were captured by microscope equipped with Olympus DP72 camera. Fluorescence images were acquired by Leica TCS SP8 microscope.

### Golgi staining

Golgi staining was performed using FD Rapid GolgiStain Kit (FD NeuroTechnologies) following the manufacturer's protocols. In brief, cerebella were dissected from 3-month-old controls and DKO mutants and incubated in premixed Solution A and B that was refreshed the next day. The cerebella were kept in the dark for 2 weeks at room temperature and then incubated in Solution C in dark for 2–7 days at 4 °C. Solution C was refreshed on the second day of incubation. The cerebella were then sectioned into 80 μm thick slices with a vibratome and mounted on gelatin-coated coverslips. The sections were then stained with premixed Solution D and E for 10 min. The sections were rinsed with water and dehydrated with ethanol series, followed by xylene and mounted on glass slides with DPX mountants. For quantification of spine density, confocal *z*-stack images with 0.3 μm step size were captured for each PC using Leica TCS SP8 microscope with 100 × oil lens. Distal dendrites were randomly chosen for analysis of spine density. The length of each dendrites and the number of dendritic spines were measured using Fiji ImageJ software^51^,^52^. Spines with enlarged spine heads (diameter of spine head ≧0.5 μm) were defined as mature spines, while elongated spines (spine length ≧1.5 μm) without enlarged spine heads were defined as immature spines.

### Transmission electron microscopy

The preparation of cerebella for electron microscopy was performed as in a previously described protocol^53^ with modifications. The 3-month-old control and DKO mice were anaesthetized and transcardially perfused with 0.9% saline (with 20 U ml^−1^ heparin), followed by 2% PFA and 2.5% glutaraldehyde in 0.1 M cacodylate buffer (pH 7.4). Cerebella were dissected and post-fixed in the same fixatives for 1 h at 4 °C. The cerebella were then sliced sagittally at 500 μm and cut into small cubes. After washing with cacodylate buffer, the samples were immersed for 1 h in 1.5% K_4_Fe(CN)_6_ with 1% OsO_4_ in cacodylate buffer, followed by 1 h in 1% OsO_4_ in cacodylate buffer at room temperature with agitation. After washing, the samples were dehydrated through an ethanol series, followed by infiltration and embedding in Spurr's resin (Polysciences). The 1 μm thick sections were cut and stained with Toluidine Blue to select suitable areas for examination. Ultrathin sections (70 nm) were cut from the selected areas and stained with uranyl acetate and lead citrate. The stained sections were viewed under Hitachi H-7650 electron microscope at 80 kV. Images were captured at 15,000 × magnification for analysis of synaptic density and at 40,000 × or 50,000 × magnifications for examination of spine morphology. For the analysis of synaptic density, only structures with clearly recognizable synaptic cleft and synaptic vesicles were counted as synapses.

### Electrophysiological analysis

Electrophysiological recordings were performed in cerebellar slices prepared from 3-week-old mice. Brains from either sex were dissected in ice-cold artificial cerebrospinal fluid (ACSF) containing the following (in mM): 2 KCl, 120 NaCl, 2 MgSO_4_, 1.2 KH_2_PO_4_, 26 NaHCO_3_, 2.5 CaCl_2_ and 11 glucose at 95% O_2_, 5% CO_2_, pH 7.4. Parasagittal cerebellar slices (250 μm) prepared using a vibratome were incubated in oxygenated ACSF at 34 °C for 1 h before recordings. The slices were transferred to a chamber mounted onto a fixed-stage upright microscope (Olympus BX51WI) superfused with ACSF at 34 °C. Aided by differential interference contrast optics, whole-cell recordings were made from PCs somatically using microelectrodes with a resistance of 6–8 MΩ. In current-clamp mode, spontaneous firings of the neurons were recorded with no current injection for a period of 3–5 min and the CVs of the interspike intervals were calculated. To record the response to hyperpolarizing and depolarizing current, 400 ms current steps ranging from −400 to 400 pA were injected to the PCs. To measure evoked EPSCs, 10 μM bicuculline was added in the ACSF. The PCs were voltage-clamped at −70 mV and paired-pulse stimuli separated by 50 ms were delivered and the evoked EPSCs were recorded. The paired-pulse stimuli consisted of square-pulse voltage stimulation each of 50 μs width and of variable intensities. For measurement of the paired-pulse ratio, a stimulation intensity was chosen (8–50 V) that evoked 40–50% of the maximum EPSC amplitude obtained. Data were captured by Digidata-pClamp system (Axon Instruments).

### Image analysis and statistics

Only age-matched controls and DKO mutants were used for group-to-group comparisons in all analyses. Images of PCs were analysed with FilamentTracer (Imaris 7.5) to measure the total dendritic length and the number of branch points. Cell density, spine density and fluorescence intensity were quantified using Fiji ImageJ^51^,^54^. Only the central focal planes of the PC proximal dendrites from the *z*-stacks of confocal images were used for the assessment of F-actin localization. For quantifications of density, random areas were used for measurement. All of the measurement was done blinded to the mouse genotypes.

Statistical analysis was performed using GraphPad Prism 5.0. Unless otherwise noted, values in all bar graphs represent mean±s.e.m. Probability values *P*<0.05 were considered as statistically significant. Normality and variance was assessed by D'Agostino and Pearson test and *F*-test, respectively. Unpaired Student's *t*-test (two-tailed) was used for comparisons involving two groups. One-way analysis of variance, followed by Turkey's multiple comparison test, was used for comparisons involving more than two groups. For data without normal distribution, Kruskal–Wallis test was applied, followed by Dunn's multiple comparison test. No statistical methods were used to predetermine sample size.

### Data availability

The data that support the findings of this study are available from the corresponding author on request. All microarray data are available in MIAME-compliant database, Center for Information Biology Gene Expression (CIBEX)^55^ with accession number CBX207 (http://cibex.nig.ac.jp/data/CBX207/).

## Additional information

**How to cite this article:** Lui, N. C. *et al*. Lhx1/5 control dendritogenesis and spine morphogenesis of Purkinje cells via regulation of Espin. *Nat. Commun.* **8**, 15079 doi: 10.1038/ncomms15079 (2017).

**Publisher's note:** Springer Nature remains neutral with regard to jurisdictional claims in published maps and institutional affiliations.

## Supplementary Material

Supplementary Information Supplementary Figures, Supplementary Tables, Supplementary Methods and Supplementary References

Supplementary Movie 1 A control mouse walks on the ledge of cage smoothly

Supplementary Movie 2 A DKO mutant mouse walks on the ledge of cage with severe body shaking

Supplementary Movie 3 A control mouse with normal gait

Supplementary Movie 4 A DKO mutant mouse with "duck-feet" hindpaws phenotype

## References

[b1] Gao Z., van Beugen B. J. & De Zeeuw C. I. Distributed synergistic plasticity and cerebellar learning. Nat. Rev. Neurosci. 13, 619–635 (2012). 22895474 10.1038/nrn3312

[b2] D'Angelo E. & Casali S. Seeking a unified framework for cerebellar function and dysfunction: from circuit operations to cognition. Front. Neural Circuits 6, 116 (2012). 23335884 10.3389/fncir.2012.00116 PMC3541516

[b3] DeZeeuw C. I. . Spatiotemporal firing patterns in the cerebellum. Nat. Rev. Neurosci. 12, 327–344 (2011). 21544091 10.1038/nrn3011

[b4] Person A. L. & Raman I. M. Purkinje neuron synchrony elicits time-locked spiking in the cerebellar nuclei. Nature 481, 502–505 (2012). 10.1038/nature10732 PMC3268051 22198670

[b5] Person A. L. & Raman I. M. Synchrony and neural coding in cerebellar circuits. Front. Neural Circuits 6, 97 (2012). 23248585 10.3389/fncir.2012.00097 PMC3518933

[b6] Sarna J. R. & Hawkes R. Patterned Purkinje cell death in the cerebellum. Prog. Neurobiol. 70, 473–507 (2003). 14568361 10.1016/s0301-0082(03)00114-x

[b7] Taroni F. & DiDonato S. Pathways to motor incoordination: the inherited ataxias. Nat. Rev. Neurosci. 5, 641–655 (2004). 15263894 10.1038/nrn1474

[b8] Carlson K. M., Andresen J. M. & Orr H. T. Emerging pathogenic pathways in the spinocerebellar ataxias. Curr. Opin. Genet. Dev. 19, 247–253 (2009). 19345087 10.1016/j.gde.2009.02.009 PMC2721007

[b9] Zhao Y. . LIM-homeodomain proteins Lhx1 and Lhx5, and their cofactor Ldb1, control Purkinje cell differentiation in the developing cerebellum. Proc. Natl Acad. Sci. USA 104, 13182–13186 (2007). 17664423 10.1073/pnas.0705464104 PMC1941824

[b10] Lein E. S. . Genome-wide atlas of gene expression in the adult mouse brain. Nature 445, 168–176 (2007). 17151600 10.1038/nature05453

[b11] Sotelo C. & Dusart I. Intrinsic versus extrinsic determinants during the development of Purkinje cell dendrites. Neuroscience 162, 589–600 (2009). 19166910 10.1016/j.neuroscience.2008.12.035

[b12] Kapfhammer J. P. Cellular and molecular control of dendritic growth and development of cerebellar Purkinje cells. Prog. Histochem. Cytochem. 39, 131–182 (2004). 15580762 10.1016/j.proghi.2004.07.002

[b13] Ikeda Y. . Spectrin mutations cause spinocerebellar ataxia type 5. Nat. Genet. 38, 184–190 (2006). 16429157 10.1038/ng1728

[b14] Knight M. A. . Spinocerebellar ataxia type 15 (sca15) maps to 3p24.2-3pter. Neurobiol. Dis. 13, 147–157 (2003). 12828938 10.1016/s0969-9961(03)00029-9

[b15] Tanaka M. Dendrite formation of cerebellar Purkinje cells. Neurochem. Res. 34, 2078–2088 (2009). 19821027 10.1007/s11064-009-0073-y

[b16] O'Brien J. & Unwin N. Organization of spines on the dendrites of Purkinje cells. Proc. Natl Acad. Sci. USA 103, 1575–1580 (2006). 16423897 10.1073/pnas.0507884103 PMC1360541

[b17] Shawlot W. & Behringer R. R. Requirement for Lim1 in head-organizer function. Nature 374, 425–430 (1995). 7700351 10.1038/374425a0

[b18] Zhao Y. . Control of hippocampal morphogenesis and neuronal differentiation by the LIM homeobox gene Lhx5. Science 284, 1155–1158 (1999). 10325223 10.1126/science.284.5417.1155

[b19] Kwan K. M. & Behringer R. R. Conditional inactivation of Lim1 function. Genesis 32, 118–120 (2002). 11857795 10.1002/gene.10074

[b20] Lewandoski M., Wassarman K. M. & Martin G. R. Zp3-cre, a transgenic mouse line for the activation or inactivation of loxP-flanked target genes specifically in the female germ line. Curr. Biol. 7, 148–151 (1997). 9016703 10.1016/s0960-9822(06)00059-5

[b21] Paylor R., Zhao Y., Libbey M., Westphal H. & Crawley J. N. Learning impairments and motor dysfunctions in adult Lhx5-deficient mice displaying hippocampal disorganization. Physiol. Behav. 73, 781–792 (2001). 11566211 10.1016/s0031-9384(01)00515-7

[b22] Zhang X. M. . Highly restricted expression of Cre recombinase in cerebellar Purkinje cells. Genesis 40, 45–51 (2004). 15354293 10.1002/gene.20062

[b23] Brooks S. P. & Dunnett S. B. Tests to assess motor phenotype in mice: a user's guide. Nat. Rev. Neurosci. 10, 519–529 (2009). 19513088 10.1038/nrn2652

[b24] Gao Y. . β-III spectrin is critical for development of purkinje cell dendritic tree and spine morphogenesis. J. Neurosci. 31, 16581–16590 (2011). 22090485 10.1523/JNEUROSCI.3332-11.2011 PMC3374928

[b25] Haag N. . The actin nucleator Cobl is crucial for Purkinje cell development and works in close conjunction with the F-actin binding protein Abp1. J. Neurosci. 32, 17842–17856 (2012). 23223303 10.1523/JNEUROSCI.0843-12.2012 PMC6621670

[b26] Georges P. C., Hadzimichalis N. M., Sweet E. S. & Firestein B. L. The yin-yang of dendrite morphology: unity of actin and microtubules. Mol. Neurobiol. 38, 270–284 (2008). 18987787 10.1007/s12035-008-8046-8

[b27] Cingolani L. A. & Goda Y. Actin in action: the interplay between the actin cytoskeleton and synaptic efficacy. Nat. Rev. Neurosci. 9, 344–356 (2008). 18425089 10.1038/nrn2373

[b28] Sekerková G. . Novel espin actin-bundling proteins are localized to Purkinje cell dendritic spines and bind the Src homology 3 adapter protein insulin receptor substrate p53. J. Neurosci. 23, 1310–1319 (2003). 12598619 10.1523/JNEUROSCI.23-04-01310.2003 PMC2854510

[b29] Hobert O. & Westphal H. Functions of LIM-homeobox genes. Trends Genet. 16, 75–83 (2000). 10652534 10.1016/s0168-9525(99)01883-1

[b30] Berger M. F. . Variation in homeodomain DNA binding revealed by high-resolution analysis of sequence preferences. Cell 133, 1266–1276 (2008). 18585359 10.1016/j.cell.2008.05.024 PMC2531161

[b31] Zheng L., Beeler D. M. & Bartles J. R. Characterization and regulation of an additional actin-filament-binding site in large isoforms of the stereocilia actin-bundling protein espin. J. Cell Sci. 127, 1306–1317 (2014). 24424026 10.1242/jcs.143255 PMC3953818

[b32] Zheng L. . The deaf jerker mouse has a mutation in the gene encoding the espin actin-bundling proteins of hair cell stereocilia and lacks espins. Cell 102, 377–385 (2000). 10975527 10.1016/s0092-8674(00)00042-8 PMC2850054

[b33] Loomis P. A. . Targeted wild-type and jerker espins reveal a novel, WH2-domain-dependent way to make actin bundles in cells. J. Cell Sci. 119, 1655–1665 (2006). 16569662 10.1242/jcs.02869 PMC2854011

[b34] Llinás R. The intrinsic electrophysiological properties of mammalian neurons: insights into central nervous system function. Science 242, 1654–1664 (1988). 3059497 10.1126/science.3059497

[b35] Pillai A., Mansouri A., Behringer R., Westphal H. & Goulding M. Lhx1 and Lhx5 maintain the inhibitory-neurotransmitter status of interneurons in the dorsal spinal cord. Development 134, 357–366 (2007). 17166926 10.1242/dev.02717

[b36] Avraham O. . Transcriptional control of axonal guidance and sorting in dorsal interneurons by the Lim-HD proteins Lhx9 and Lhx1. Neural Dev. 4, 21 (2009). 19545367 10.1186/1749-8104-4-21 PMC2704203

[b37] Sekerková G., Zheng L., Mugnaini E. & Bartles J. R. Differential expression of espin isoforms during epithelial morphogenesis, stereociliogenesis and postnatal maturation in the developing inner ear. Dev. Biol. 291, 83–95 (2006). 16413524 10.1016/j.ydbio.2005.12.021 PMC2586395

[b38] Loomis P. A. . Espin cross-links cause the elongation of microvillus-type parallel actin bundles in vivo. J. Cell Biol. 163, 1045–1055 (2003). 14657236 10.1083/jcb.200309093 PMC2173610

[b39] Bartles J. R., Zheng L., Li A., Wierda A. & Chen B. Small espin: a third actin-bundling protein and potential forked protein ortholog in brush border microvilli. J. Cell Biol. 143, 107–119 (1998). 9763424 10.1083/jcb.143.1.107 PMC2132824

[b40] Choi J. . Regulation of dendritic spine morphogenesis by insulin receptor substrate 53, a downstream effector of Rac1 and Cdc42 small GTPases. J. Neurosci. 25, 869–879 (2005). 15673667 10.1523/JNEUROSCI.3212-04.2005 PMC6725612

[b41] Burette A. C., Park H. & Weinberg R. J. Postsynaptic distribution of IRSp53 in spiny excitatory and inhibitory neurons. J. Comp. Neurol. 522, 2164–2178 (2014). 24639075 10.1002/cne.23526 PMC3997599

[b42] Scita G., Confalonieri S., Lappalainen P. & Suetsugu S. IRSp53: crossing the road of membrane and actin dynamics in the formation of membrane protrusions. Trends Cell Biol. 18, 52–60 (2008). 18215522 10.1016/j.tcb.2007.12.002

[b43] Sekino Y., Kojima N. & Shirao T. Role of actin cytoskeleton in dendritic spine morphogenesis. Neurochem. Int. 51, 92–104 (2007). 17590478 10.1016/j.neuint.2007.04.029

[b44] Sekerková G., Richter C.-P. & Bartles J. R. Roles of the espin actin-bundling proteins in the morphogenesis and stabilization of hair cell stereocilia revealed in CBA/CaJ congenic jerker mice. PLoS Genet. 7, e1002032 (2011). 21455486 10.1371/journal.pgen.1002032 PMC3063760

[b45] McKay B. E. & Turner R. W. Physiological and morphological development of the rat cerebellar Purkinje cell. J. Physiol. 567, 829–850 (2005). 16002452 10.1113/jphysiol.2005.089383 PMC1474219

[b46] Windhorst S. . Inositol-1,4,5-trisphosphate 3-kinase A regulates dendritic morphology and shapes synaptic Ca2+ transients. Cell. Signal. 24, 750–757 (2012). 22120525 10.1016/j.cellsig.2011.11.010

[b47] Terauchi A. . Distinct FGFs promote differentiation of excitatory and inhibitory synapses. Nature 465, 783–787 (2010). 20505669 10.1038/nature09041 PMC4137042

[b48] Dreos R., Ambrosini G., Cavin Périer R. & Bucher P. EPD and EPDnew, high-quality promoter resources in the next-generation sequencing era. Nucleic Acids Res. 41, D157–D164 (2013). 23193273 10.1093/nar/gks1233 PMC3531148

[b49] Vernes S. C. . Foxp2 regulates gene networks implicated in neurite outgrowth in the developing brain. PLoS Genet. 7, e1002145 (2011). 21765815 10.1371/journal.pgen.1002145 PMC3131290

[b50] Nelson J. D., Denisenko O. & Bomsztyk K. Protocol for the fast chromatin immunoprecipitation (ChIP) method. Nat. Protoc. 1, 179–185 (2006). 17406230 10.1038/nprot.2006.27

[b51] Schindelin J. . Fiji: an open-source platform for biological-image analysis. Nat. Methods 9, 676–682 (2012). 22743772 10.1038/nmeth.2019 PMC3855844

[b52] Orlowski D. & Bjarkam C. R. A simple reproducible and time saving method of semi-automatic dendrite spine density estimation compared to manual spine counting. J. Neurosci. Methods 208, 128–133 (2012). 22595026 10.1016/j.jneumeth.2012.05.009

[b53] Harris K. M. & Stevens J. K. Dendritic spines of rat cerebellar Purkinje cells: serial electron microscopy with reference to their biophysical characteristics. J. Neurosci. 8, 4455–4469 (1988). 3199186 10.1523/JNEUROSCI.08-12-04455.1988 PMC6569567

[b54] Schneider C. A., Rasband W. S. & Eliceiri K. W. NIH Image to ImageJ: 25 years of image analysis. Nat. Methods 9, 671–675 (2012). 22930834 10.1038/nmeth.2089 PMC5554542

[b55] Ikeo K., Ishi-i J., Tamura T., Gojobori T. & Tateno Y. CIBEX: Center for information biology gene EXpression database. C. R. Biol. 326, 1079–1082 (2003). 14744116 10.1016/j.crvi.2003.09.034

