# Supplementary material for: Lhx1/5 control dendritogenesis and spine morphogenesis of Purkinje cells via regulation of Espin
Source: Nat Commun. 2017 May 18;8:15079. doi: 10.1038/ncomms15079 (PMC5454373; doi:10.1038/ncomms15079)
Supplement: Supplementary Information — Supplementary Figures, Supplementary Tables, Supplementary Methods and Supplementary References [file ncomms15079-s1.pdf]

Supplementary Information

Supplementary Figure 1

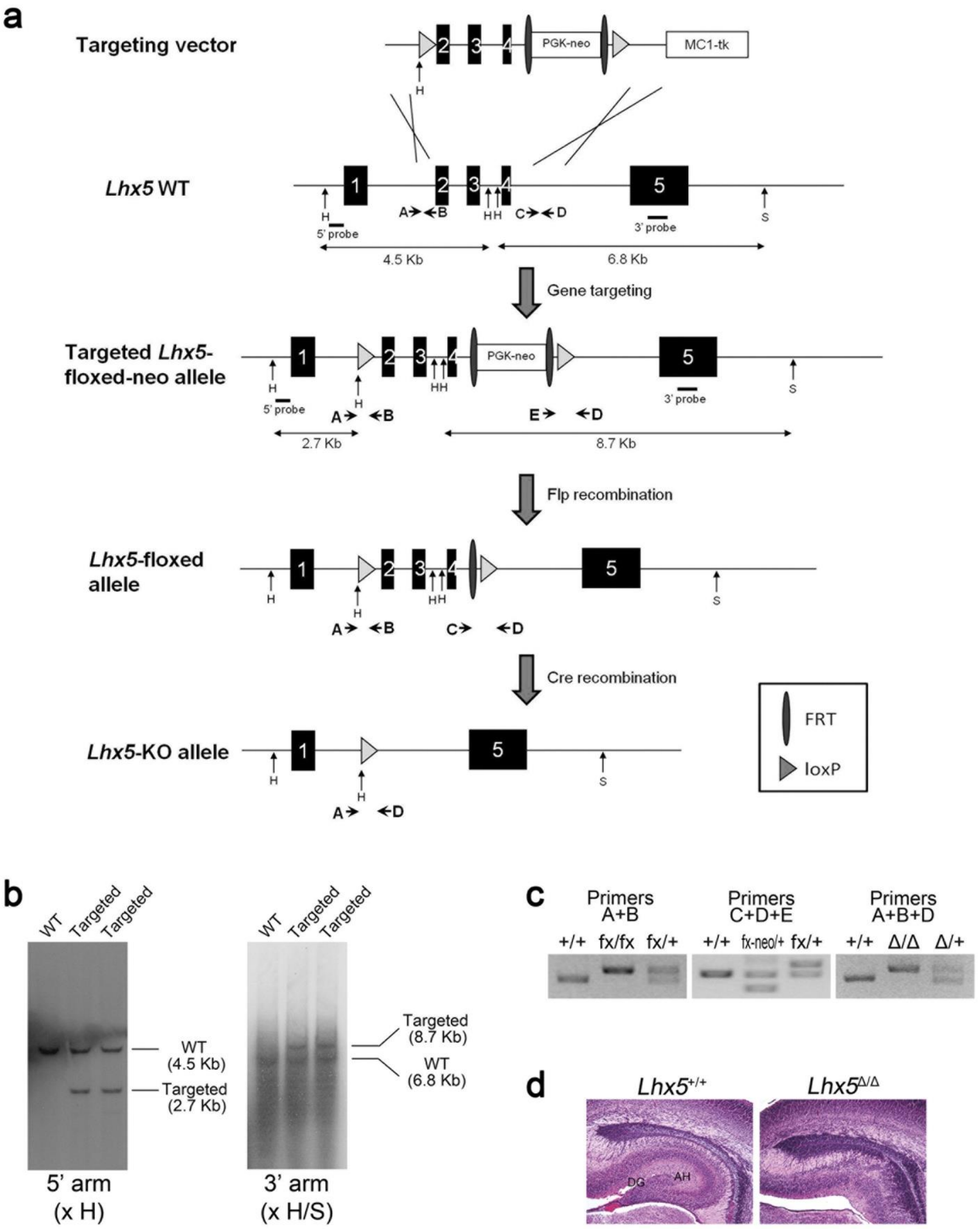

Supplementary Figure 1. Generation of *Lhx5* conditional null alleles. (**a**) Genomic organization of the mouse *Lhx5* gene with five exons. The gene targeting vector was generated by inserting two *lox*P sites (grey triangles) flanking exons 2 to 4 of *Lhx5* and a *PGKneobpA* cassette flanked by *FRT* sites (dark ovals) just 3' to exon 4. A thymidine kinase (*MC1-tk*) selection cassette was added at the 3' end of homology. The *FRT* flanked *PGKneobpA* cassette was removed by crossing the *Lhx5*-floxed-neo mice with FLPeR mice. Locations of PCR primers, external 5' and 3' Southern probes and sizes of corresponding restriction enzyme digested DNA fragments are shown. (**b**) Representative Southern blots showing correct homologous recombination in two ES cell clones. Genomic DNA was digested with *Hind*III (for 5' probe) or *Hind*III and *Sca*I (for 3'probe). H: *Hind*III; S: *Sca*I. (**c**) PCR genotyping of wildtype (+) versus *Lhx5* floxed (*fx*) allele (using primers A and B), wild type versus *Lhx5* floxed-neo (*fx*-neo) and *Lhx5* floxed allele (using primers C, D and E) and wild type versus *Lhx5* null (Δ) allele (using primers A, B and D) were shown. (**d**) Absence of both dentate gyrus (DG) and Ammon's horn (AH) in the hippocampus of *Lhx5* recombined homozygous mutant at P0 validated the functionality of the *Lhx5* conditional allele. Genotyping primer sequences were: A: 5' – AAG GGA AAG AGG GCT TTG GAG TGA – 3'; B: 5' – AGA GTT TGG CCT GGA GTG AAG – 3'; C: 5' – CCG CCT CAT TGC CTA ATT TCC ACT G – 3'; D: 5' – TCG GCG GTC CAG AGA AAT GCA AAT – 3'; E: 5' – TGC GGA ACC CTT CGA AGT TCC TAT – 3'. PCR product sizes: Wild type (WT): 537 bp; *fx*: 623 bp (for primers A+B); WT: 455 bp; *fx*-neo: 338 bp; *fx*: 559 bp (for primers C+D+E); WT: 537 bp; null: 615 bp (for primers A+B+D).

Supplementary Figure 2

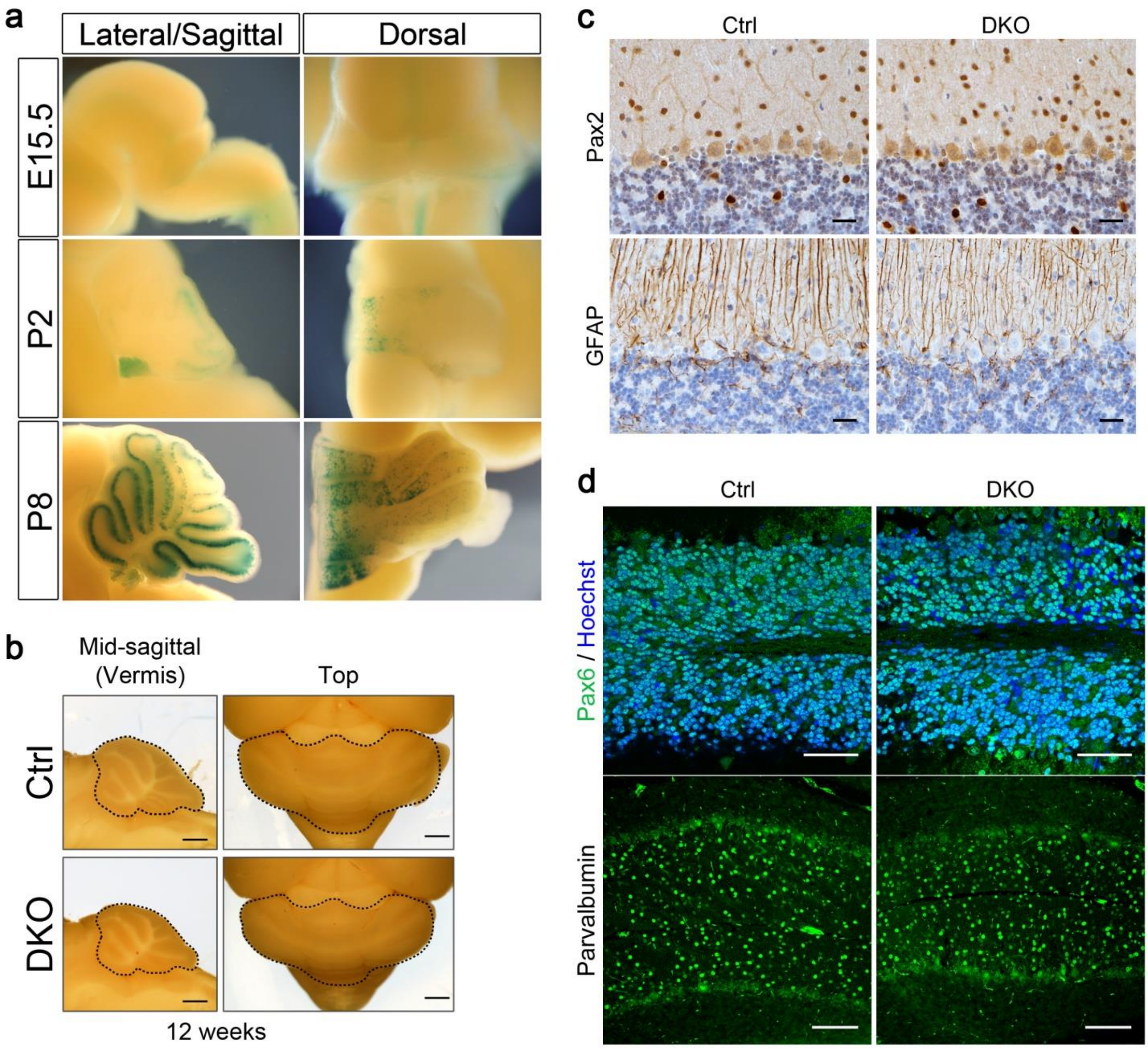

Supplementary Figure 2. *Pcp2-Cre* is specifically expressed in postnatal PCs and conditional inactivation of *Lhx1/5* does not affect other cerebellar cells. (**a**) Whole mount X-gal staining for β-galactosidase activity in brains of *Pcp2-Cre*; *R26R/+* mice. No Cre reporter expression was detected in embryonic cerebellum at E15.5. β-galactosidase activity was first detected in some PCs at the medial vermis at P2. At P8, most of the PCs at the vermis showed Cre reporter expression. (**b**) Images of cerebella from the control and the DKO mutant revealed the surface area (outlined by the

dotted lines) of the cerebellum were reduced in the DKO mutant. Scale bars, 1mm. (**c**) Other GABAergic interneurons (labeled by Pax2) and Bergmann glial fibres (labeled by GFAP) had comparable morphology between the control and the DKO mutant. Scale bars, 20 μm. (**d**) Granule cells (labeled by Pax6) remained normal in the DKO mutant, but GABAergic interneurons in the molecular layer (labeled by Parvalbumin) packed more densely in the DKO mutant. Scale bars, (upper panels) 50 μm; (lower panels) 100 μm.

Supplementary Figure 3

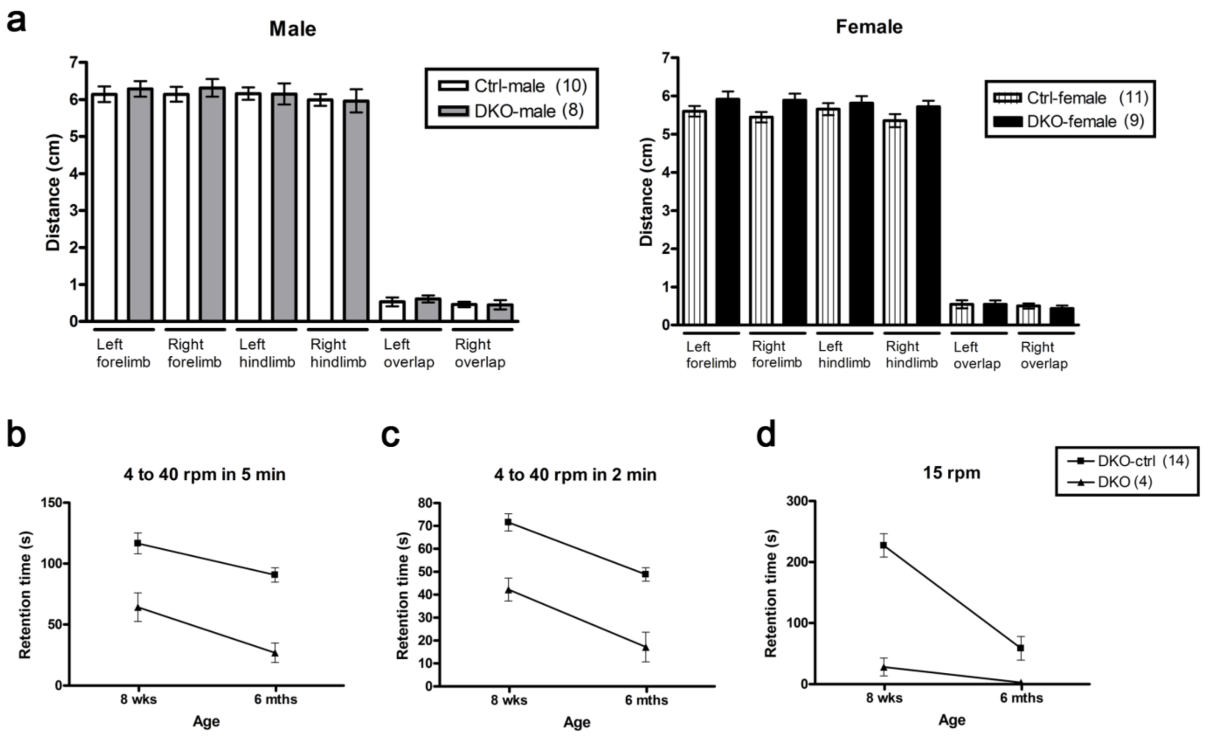

Supplementary Figure 3. ***Lhx1/5*** **DKO mutant mice display non-progressive motor deficits.**

(**a**) Bar graphs showing there were no significant differences in both stride lengths and forelimb-hindlimb overlapping between the DKO mutants and the controls of both sexes. (**b-d**) Retention time before falling from an accelerating rotarod from 4 to 40 rpm in 5 min (**b**) or in 2 min (**c**) and stationary rotarod at 15 rpm (**d**) for 8 week and 6 month old mice. The performance of both the DKO mutants and the controls declined from 8 weeks to 6 months and the reduction in retention time (i.e. the slope) was similar between the DKO mutants and the controls, suggesting that there was no significant progressive deterioration in motor coordination in the DKO mutants. The numbers in the brackets indicate the number of mice tested. Values are shown as mean ± s.e.m.

Supplementary Figure 4

>gi|372099106:152136281-152137366 *Mus musculus* strain C57BL/6J chromosome 4, GRCm38.p2 C57BL/6J (reverse complimentary)

AGTCCAGGGCCCTGTCCCAGATAATTCAACGCTCACCTTGAGGCAGTAATAGAAAAGGAAAAAGATGGGTCTCACA
GCCCCTCTCAGAGGGATGCTATCTCCAGGGCCCAGCCCAGCCATGAGGAAGTCTTCCAGATGAAGCCGGAGGAAA
GAAGGCCAATAGGCTTGCTCTGCTTGCTAGTGTGTAGGTGATCCTGGCTGGGTTGTGAACAGTACAGCAGGGCCTA
GGGGTCAAGCAAGCTGAAAGGTTAGGAGATGAACTTCCAGAGAGGGTCTCCCAGAGTCAGGGCCCCAAGAGCCTG
CCCGGCTCCAGCTCCTGATGCTGAGCCAGTCATAAGCCACTGTTGAATTCTGACACTGGTAAGCTCTCTGGCCTGG
GCTCCACAGGTGTGACCTAGTTCATCTTTGCCTATGATTGGCACTTCATGATTAAGGCATGTATTAGGTGGCATGCA
GTGGTCTGTCACCAACTTTCAACACCTTTGCAAACACTGTTACTCCTACTTACAGATGAAGCAATTCAGGCGCAGAA
GATACTGGTTCATAATCTCATAGCACAGTAAGGATGGGAAAGTAAAGGTATTATTCCAGGATCCAAAGTCTGTGCCC
TGTGTTCCAGCTTCCATAGGCTACAGAAGTCAGGGCAGAGTCCCAGTGCTGCCCCCTAGTGCCAATCAAGAAAAGA
GCAACTGCAGGTTGTCTCTCTCCCCCCCGCCCCCACTCTTCCAGCCCCCCCCAAAGTCATCTTCTGGGGCTCCCTT
TCCTACAGGGTAGACAACAGGGCCTGGAGTCCTTGAAGTGAATGGGATAGGCAGCAGGATCTGGCCCCCTCACCC
AGGCAGGCTCAGCAGCAGCAATGACAGATCCAAGCCACCAAGGTAGCTGGCCCAGAAAAGGCAAACAC***TAATTA***C
AGAGCCAATAAGCATGGCTGCTGAGCCGTTACCTGGTGCCAGCCTGCTTCCCTCTCCCAGGGGGCCTGTGACACT
TCTCAGGCAGGGAGGGGCAAGGGGCAGGGAGGAGTAGCTGTGGGCAGGAGGCTATAGAGAGGAGGAGGAGGAG
CAGGCTTGCCGGTAGCAGGGACAGCAT

Supplementary Figure 4. *Espn* promoter sequence used for the SEAP reporter assay and ChIP-PCR experiment. The sequence highlighted grey indicates the predicated promoter sequence retrieved from EPD. The sequence in blue indicates the predicated binding site of Lhx1/5 homeodomain. The underlined parts are the primer sequences used for ChIP-PCR.

Supplementary Figure 5

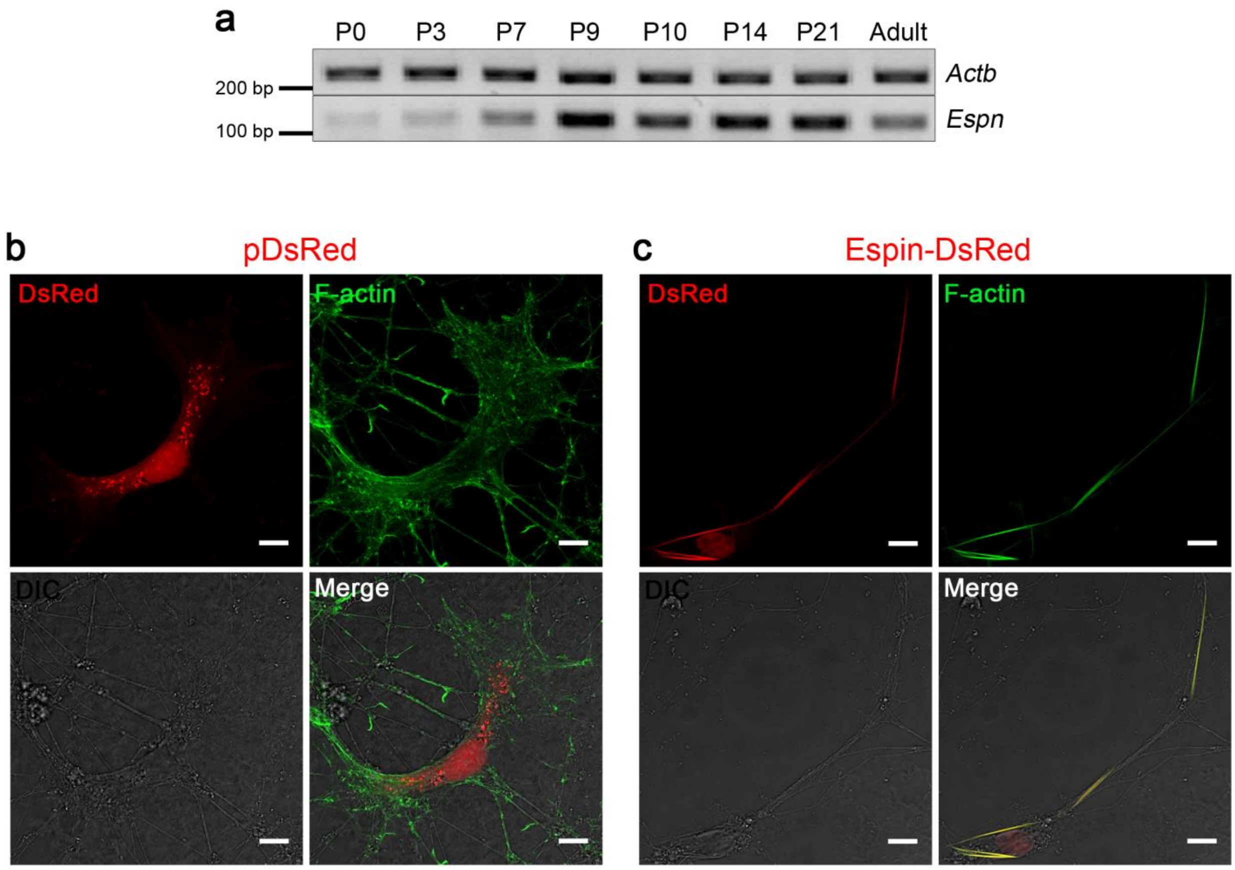

Supplementary Figure 5. Espin expresses in postnatal cerebellum and regulates F-actin organization in cells. (**a**) Semi-quantitative RT-PCR revealed that *Espn* started to robustly express in cerebellum from P9 onwards. (**b, c**) Mouse fibroblast cells were transfected with pDsRed (**b**) or Espin-DsRed (**c**) constructs. The cells were stained with Alexa Fluor 488 phalloidin to visualize the F-actin cytoskeleton. Espin was highly co-localized with F-actin and the organization of F-actin was controlled by the expression of Espin. With Espin expressed, F-actin was organized into coarse bundles which supported the cells to form long neurite-like structure. All scale bars, 10 μm.

Supplementary Figure 6

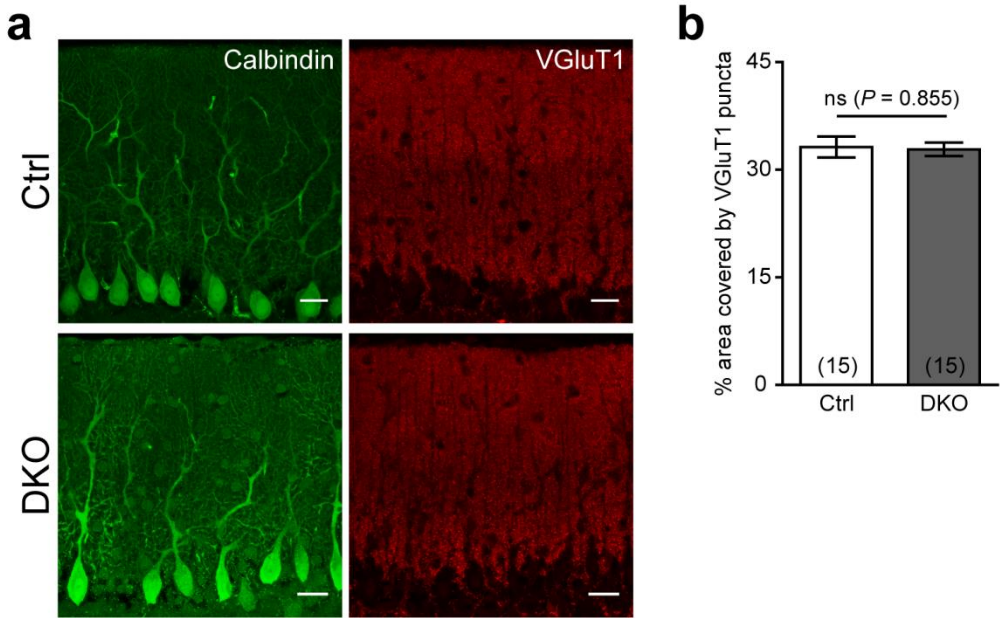

Supplementary Figure 6. PF-PC innervations in the DKO mutants. (**a**) Immunostaining of calbindin (green) and VGluT1 (red) showing no observable difference between the controls (top) and the DKO mutants (bottom). (**b**) Quantification of the percentage area of molecular layer covered by PFs (VGluT1 puncta) showed that there were no significant difference in the number of PFs between the controls and the DKO mutants. The numbers in the brackets indicate the number of sections tested. N = 3 mice per group. Scale bars, 20 μm.

Supplementary Figure 7

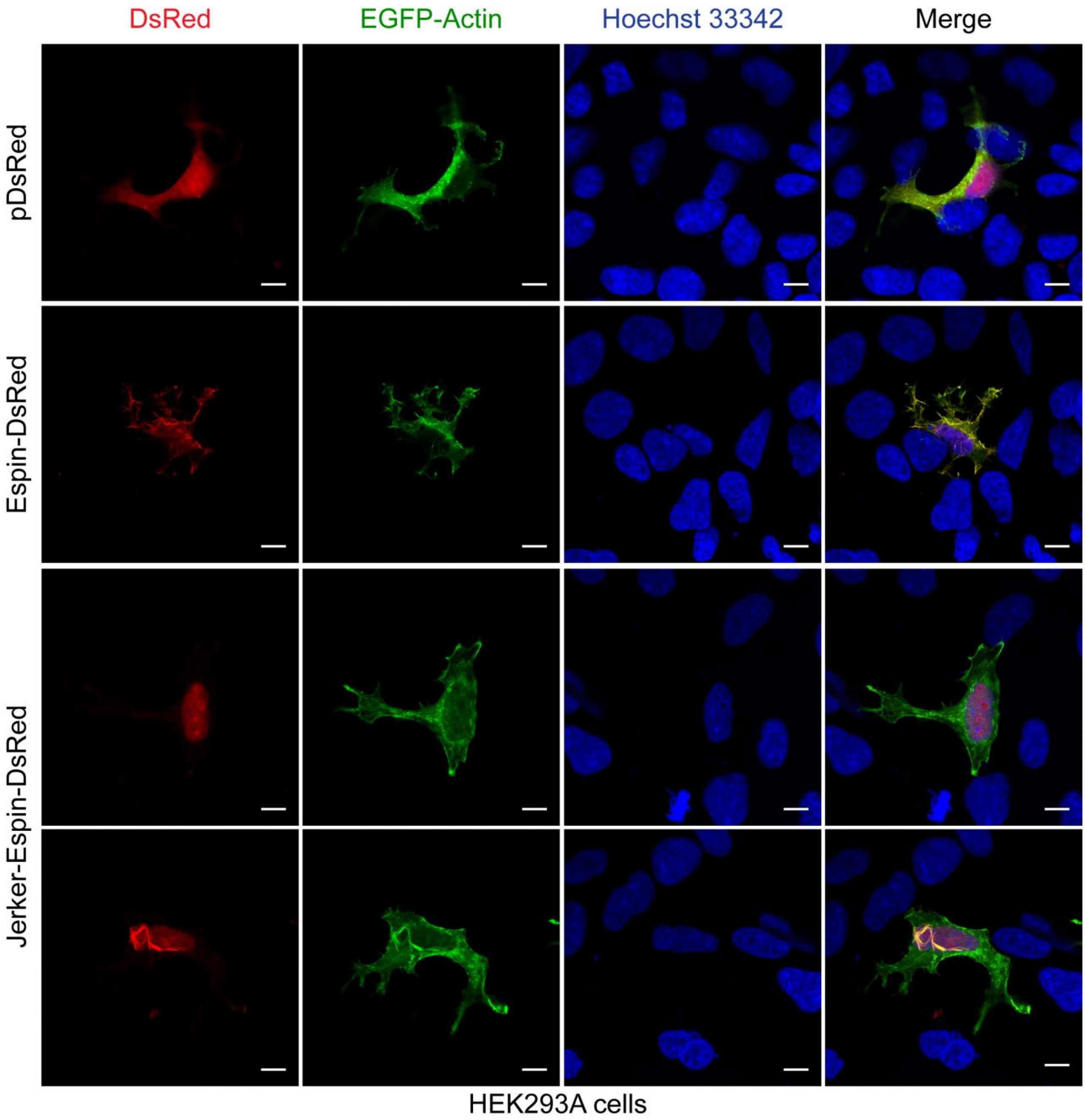

Supplementary Figure 7. Espin with *jerker* mutation retains and bundles F-actin inside the nucleus. HEK293A cells transfected with pDsRed, Espin-DsRed and jerker-Espin-DsRed respectively. Normally, Espin bundled and organized F-actin in the cytoplasmic region of the cells. With *jerker* mutation, mutated Espin became retained in the nuclei of the cells and bundled F-actin inside the nuclei. Scale bars, 10 µm.

Supplementary Figure 8

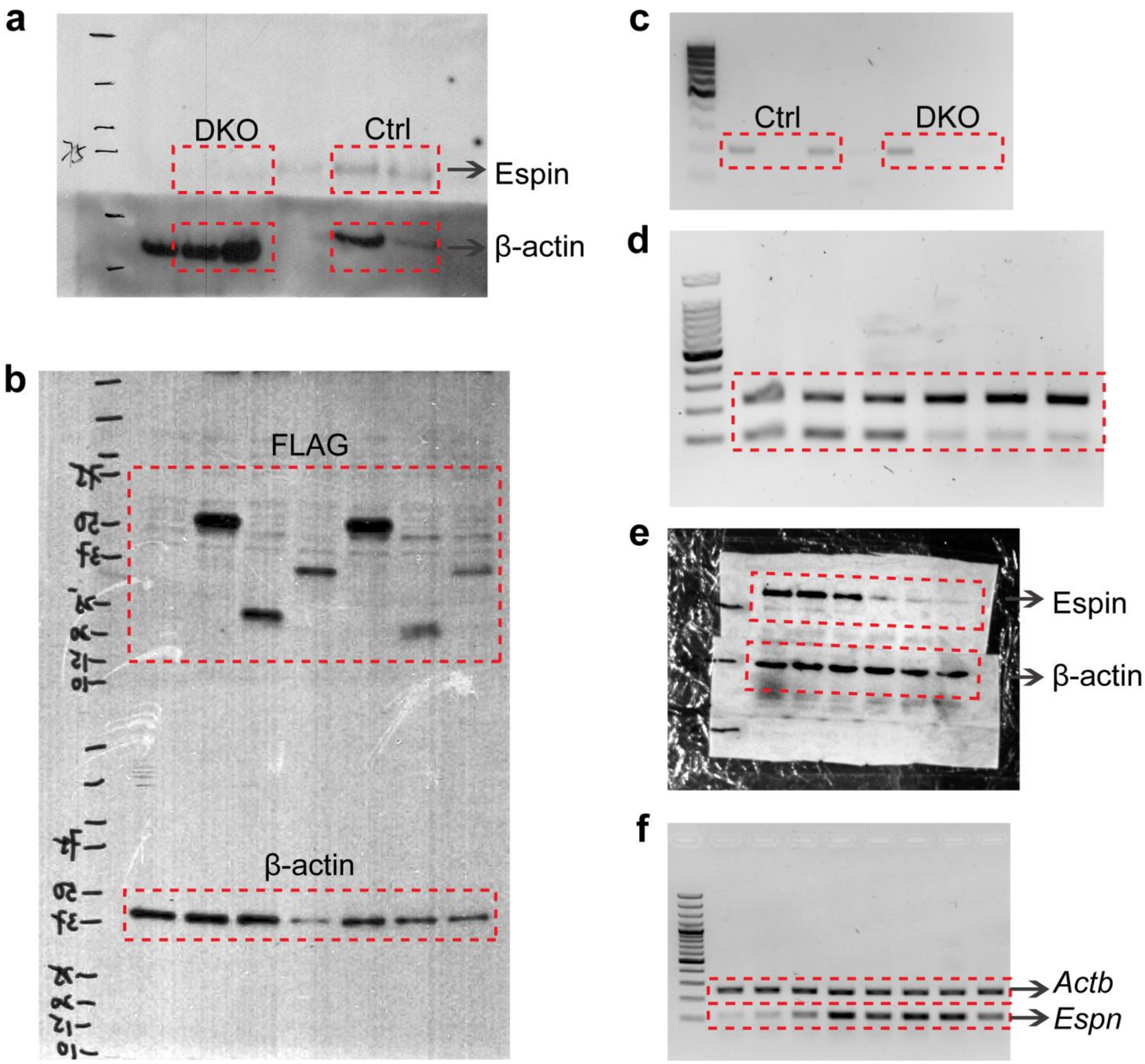

Supplementary Figure 8. Original blots or gels. (**a**) Original Western blot of Fig. 3b. (**b**) Original Western blot of Fig. 3d. (**c**) Original gel photo of Fig. 3g. (**d**) Original gel photo of Fig. 4a. (**e**) Original Western blot of Fig. 4b. (**f**) Original gel photo of Supplementary Fig. 5a. Red boxes indicate the portions used for the figures.

Supplementary Table 1. Genes with >5-fold up-regulation in the *Lhx1/5* DKO mutant cerebellum compared with the control

Fold change	Gene Symbol	Gene Name	Gene ID
29.69	Htr1a	5-hydroxytryptamine (serotonin) receptor 1A	4716
23.22	Gabra5	gamma-aminobutyric acid (GABA) A receptor, subunit alpha 5	273114
18.90	Mcf2	mcf.2 transforming sequence	93796
17.81	Myo5b	myosin VB	260098
15.54	Gata3	GATA binding protein 3	313866
14.94	Wdr72	WD repeat domain 72	335289
14.77	Prlr	prolactin receptor	10516
14.43	Irs4	insulin receptor substrate 4	261591
14.43	Crhbp	corticotropin releasing hormone binding protein	316614
13.98	Sfrp1	secreted frizzled-related protein 1	281691
12.28	Irx4	Iroquois related homeobox 4 (Drosophila)	103784
11.88	Prlr	prolactin receptor	10516
11.69	Cbln4	cerebellin 4 precursor protein	40555
10.74	Bcl11b	B-cell leukemia/lymphoma 11B	392694
10.31	Vgll3	vestigial like 3 (Drosophila)	25670
9.21	Pkp1	plakophilin 1	4494
9.18	Fam19a2	family with sequence similarity 19, member A2	51939
9.13	Rxfp3	relaxin family peptide receptor 3	209312
8.58	Fam159b	family with sequence similarity 159, member B	159995
7.50	B230219N05Rik	RIKEN cDNA B230219N05 gene	447142
6.80	1700030C10Rik	RIKEN cDNA 1700030C10 gene	273972
6.55	1700001L19Rik	RIKEN cDNA 1700001L19 gene	272795
6.25	Gm5797	predicted gene 5797	422841
5.94	Elfn1	leucine rich repeat and fibronectin type III, extracellular 1	237102
5.90	Gpr26	G protein-coupled receptor 26	208740
5.83	Bcl11b	B-cell leukemia/lymphoma 11B	392694
5.80	Zfhx3	zinc finger homeobox 3	416972
5.79	Olfr799	olfactory receptor 799	377752
5.79	Gm3161	predicted gene 3161	464268
5.75	Gm8267	predicted gene 8267	387002
5.74	Tex15	testis expressed gene 15	280624
5.67	B230209E15Rik	RIKEN cDNA B230209E15 gene	333898
5.64	1500015O10Rik	RIKEN cDNA 1500015O10 gene	50109
5.56	Chrna5	cholinergic receptor, nicotinic, alpha polypeptide 5	103778
5.55	Necab2	N-terminal EF-hand calcium binding protein 2	356184
5.50	Acox2	acyl-Coenzyme A oxidase 2, branched chain	28700
5.43	Tnfsf11	tumor necrosis factor (ligand) superfamily, member 11	249221
5.38	Onecut3	one cut domain, family member 3	250572
5.37	Cox6a2	cytochrome c oxidase, subunit VI a, polypeptide 2	43824
5.01	Svopl	SV2 related protein homolog (rat)-like	197449

Supplementary Table 2. Genes with >5-fold down-regulation in the *Lhx1/5* DKO mutant cerebellum compared with the control

Fold change	Gene Symbol	Gene Name	Gene ID
35.61	Prl2c5	prolactin family 2, subfamily c, member 5	392548
24.68	2410124H12Rik	RIKEN cDNA 2410124H12 gene	444318
22.22	Prl2c3	prolactin family 2, subfamily c, member 3	457984
20.00	Prl2c3	prolactin family 2, subfamily c, member 3	457984
19.83	Prl2c5	prolactin family 2, subfamily c, member 5	392548
17.88	Fgf7	fibroblast growth factor 7	330557
16.73	Corin	corin	332425
15.52	Pkp3	plakophilin 3	350037
11.87	Nek2	NIMA (never in mitosis gene a)-related expressed kinase 2	33773
11.00	Hes3	hairy and enhancer of split 3 (Drosophila)	3516
9.78	Olfr1056	olfactory receptor 1056	377792
8.37	1700023E05Rik	RIKEN cDNA 1700023E05 gene	151092
8.30	Nrk	Nik related kinase	22367
7.75	Atp2a3	ATPase, Ca++ transporting, ubiquitous	6306
7.71	A730090H04Rik	RIKEN cDNA A730090H04 gene	442212
7.70	BB217526	expressed sequence BB217526	440693
7.58	B3gnt5	UDP-GlcNAc:betaGal beta-1,3-N-acetylglucosaminyltransferase 5	33935
7.41	Itpka	inositol 1,4,5-trisphosphate 3-kinase A	65337
7.28	Stk17b	serine/threonine kinase 17b (apoptosis-inducing)	25559
6.16	Doc2b	double C2, beta	5137
5.92	Cep76	centrosomal protein 76	111928
5.71	Tnc	tenascin C	454219
5.70	Fam107b	family with sequence similarity 107, member B	277864
5.52	Slc1a6	solute carrier family 1 (high affinity aspartate/glutamate transporter), member 6	6257
5.42	Stac	src homology three (SH3) and cysteine rich domain	1414
5.38	Cd28	CD28 antigen	255003
5.32	Dlx4	distal-less homeobox 4	439766
5.26	Espn	espin	264215
5.02	Itpr1	inositol 1,4,5-triphosphate receptor 1	227912

Supplementary Table 3. List of primers for used for cloning, RT-PCR and mutagenesis

Primer name	Sequence (5' to 3')^	Purpose
Espn-qRT-F	CTGCCTGGAGACGAGACATT	Forward primer for RT-PCR of *Espn*
Espn-qRT-R	GACTGTTCTTTCGCCCTCTG	Reverse primer for RT-PCR of *Espn*
Actb-RT-1F	TAAAGACCTCTATGCCAACACAGT	Forward primer for RT-PCR of *Actb*
Actb-RT-1R	CACGATGGAGGGGCCGGACTCATC	Reverse primer for RT-PCR of *Actb*
Gadph-qRT-F	CATGGCCTTCCGTGTTCCTA	Forward primer for RT-PCR of *Gapdh*
Gadph-qRT-R	CCTGCTTCACCACCTTCTTGAT	Reverse primer for RT-PCR of *Gapdh*
Espn-ISH-F	TTTgaattcTCCCAGCCTGAGTCACCGCAG	Forward primer for cloning of *Espn* probes for *in situ* hybridization
Espn-ISH-R	TTTaagcttTGCGTCAGCAGCTTGGGCAC	Reverse primer for cloning of *Espn* probes for *in situ* hybridization
Espn-SEAP-EmF1	TTTctcgagGACTCAGGCTCCGGTGAATGTGTCC	Forward primer for cloning of *Espn* promoter
Espn-SEAP-EmR1	TTTaagcttATGCTGTCCCTGCTACCGGCAAG	Reverse primer for cloning of *Espn* promoter and for ChIP-PCR of *Espn* promoter
Espn-pm-SEAP -mut-1F	TTTgatatcCAGAGCCAATAAGCATGGCTG	Forward primer for mutagenesis of *Espn* promoter and for ChIP-PCR of *Espn* promoter
Espn-pm-SEAP -mut-1R	TTTgatatcGTGTTTGCCTTTTCTGGGCC	Reverse primer for mutagenesis of *Espn* promoter
Lhx1-FL-FLAG-F	TTTggatccATGGTGCACTGTGCGGGCTGCAAAA	Forward primer for cloning of FLAG-Lhx1-FL/LIM
Lhx1-FL-FLAG-R	TTTaagcttCCACACGGCTGCCTCGTTCATTTCA	Reverse primer for cloning of FLAG-Lhx1-FL
Lhx1-LIM-FLAG-R	TTTaagcttATCCTCTTTACAAACGAACTTGTTCTCG	Reverse primer for cloning of FLAG-Lhx1-LIM
Lhx1-Hom-FLAG-F	TTTggatccAAACGTAGGGGACCCCGGACCACGAT	Forward primer for cloning of FLAG-Lhx1-Hom
Lhx1-Hom-FLAG-R	TTTaagcttTTTCATCCTTCGCTCCTTGGAGC	Reverse primer for cloning of FLAG-Lhx1-Hom
Lhx5-FL-FLAG-F	TTTgtcgacTATGATGGTGCACTGTGCTGGCT	Forward primer for cloning of FLAG-Lhx5-FL/LIM
Lhx5-FL-FLAG-R	TTTctcgagCTTACCATACGGCCGCTTCGTTGA	Reverse primer for cloning of FLAG-Lhx5-FL/Hom
Lhx5-LIM-FLAG-R	GTCCTGTAACGGATCCTGGAGGTC	Reverse primer for cloning of FLAG-Lhx5-LIM
Lhx5-Hom-FLAG-F	TTTgaattcggGACCTCCAGGATCCGTTACAGGAC	Forward primer for cloning of FLAG-Lhx5-Hom
Espin-DsRed-F	TTTgaattcggATGGGCAATAGCTTGGAACACCGAG	Forward primer for cloning of Espin-DsRed
Espin-DsRed-R	TTTgtcgacTTCGGGATCTCACCCTTCTTCAAGATGAC	Reverse primer for cloning of Espin-DsRed
Actb-EGFP-C1-F	TTTctcgagctATGGATGACGATATCGCTGCGC	Forward primer for cloning of Actin-EGFP
Actb-EGFP-C1-R	TTTgcggccgcCCTAGAAGCACTTGCGGTG	Reverse primer for cloning of Actin-EGFP
jerker-DsRed-F	AGACATTCTTTGGAAGAAGCTGGAGGAGG	Forward primer for mutagenesis of Espin-DsRed
jerker-DsRed-R	GTCTCCAGGCAGGCAGGC	Reverse primer for mutagenesis of Espin-DsRed
Espin-mut-FL-R	TTTgtcgactgGTGTCCGCAGCTTCTCCGACT	Reverse primer for cloning of jerker-Espin-DsRed

^Notes: Sequences in small letters represent the restriction enzyme sites for the purpose of cloning. Sequences in red represent silent mutation for the purpose of minimizing secondary structures within the primer or dimers between primer pairs.

Supplementary Table 4. List of primary antibodies used for immunostaining

Antibody	Dilution	Supplier	Catalogue number
Mouse anti-Calbindin D-28K	1:200	Sigma	C9848
Rabbit anti-GFAP	1:2,000	DakoCytomation	Z0334
Rabbit anti-IP3R1	1:2,000	Millipore	ABS55
Rabbit anti-LIM1	1:100	Abcam	ab14554
Mouse anti-Parvalbumin	1:1,000	Millipore	MAB1572
Rabbit anti-Pax2	1:100	Invitrogen	71-6000
Rabbit anti-Pax6	1:500	Millipore	AB2237
Rabbit anti-VGluT1	1:1,000	Synaptic Systems	135303
Guinea pig anti-VGluT2	1:1,000	Millipore	AB2251

Supplementary Methods

Generation of *Lhx5* conditional knockout allele by gene targeting in mouse embryonic stem (ES) cells

Lhx5 conditional knockout allele (floxed) (*fx*) was generated (Supplementary Fig. 1) following a previously described protocol[1]. Two *lox*P sites were introduced to flank exons 2 and 4 of the mouse *Lhx5* gene. The linearized targeting construct was electroporated into 129S6/B6-F1 hybrid mouse ES cell line G4 that is obtained from ES cell core facility at Samuel Lunenfeld Research Institute of Mount Sinai Hospital in Toronto, Canada[2]. G418 and FIAU double-resistant ES cell clones were screened and analyzed by Southern blot, using 5' and 3' external probes. Correctly targeted ES cell clones were injected into C57BL6 blastocysts following standard procedures. After confirmation of germline transmission of the *Lhx5 fx*-neo allele, mice were crossed with *FLPeR* mice[3] to remove the *FRT*-flanked neomycin resistance expression cassette, creating the *Lhx5 fx* mouse. Tail tips of pups were collected for DNA extraction and genotyped by PCR.

Gene expression microarray analysis

Male controls (*Pcp2*-Cre/+; $Lhx1^{fx/+}$; $Lhx5^{fx/fx}$) and *Lhx1/5* DKO mutants (n = 3 mice for each genotype) were sacrificed at 12 weeks of age. Total cerebellum RNA was extracted by TRIzol reagent (Life Technologies) and subjected to gene expression profiling using the Agilent microarray platform (Agilent Technologies). The microarray chips contained whole mouse genome oligonucleotides in

4×44K format. Labeling reactions were performed using 1 μg total RNA with Cy5-CTP from Agilent Low RNA Input Linear Amplification and Labeling Kit PLUS according to the manufacturer's protocol. After hybridization at 65 ℃ for 17 h, the microarray chip was scanned using an Agilent Microarray Scanner G2565BA (Agilent). Data was acquired by Feature Extraction 10.5 and analyzed by GeneSpring GX 11.0 software (Agilent). The signal intensity data were background subtracted, control spot calibrated, and intra-array Lowess normalized. Relative signal intensities and logarithmic ratios were calculated and compared. To calculate significant differentially expressed genes, one-way ANOVA and pairwise bootstrap statistics were performed to identify significantly expressed genes with P values < 0.05.

Supplementary References

1. Liu, P., Jenkins, N. a &Copeland, N. G. A highly efficient recombineering-based method for generating conditional knockout mutations. *Genome Res.* **13,** 476–84 (2003).

2. Vintersten, K. *et al.* Mouse in red: red fluorescent protein expression in mouse ES cells, embryos, and adult animals. *Genesis* **40,** 241–6 (2004).

3. Farley, F. W., Soriano, P., Steffen, L. S. &Dymecki, S. M. Widespread recombinase expression using FLPeR (flipper) mice. *Genesis* **28,** 106–10 (2000).
